# Diversity of the genus *Cryobacterium* and proposal of 19 novel species isolated from glaciers

**DOI:** 10.3389/fmicb.2023.1115168

**Published:** 2023-03-20

**Authors:** Qing Liu, Lei-Lei Yang, Yu-Hua Xin

**Affiliations:** China General Microbiological Culture Collection Center (CGMCC), Institute of Microbiology, Chinese Academy of Sciences, Beijing, China

**Keywords:** *Cryobacterium*, glacier-inhabiting bacteria, cold adaptation, psychrophilic, taxonomy

## Abstract

The bacterial genus *Cryobacterium* includes at present 14 species that live in cryospheric environments. In this study, we analyzed 101 genomes of *Cryobacterium* with pure cultures obtained from GenBank. They could be classified into 44 species based on average nucleotide identity (ANI) analysis, showing the diversity of *Cryobacterium*. Among these, 19 strains in our laboratory were isolated from the glacier samples in China. The pairwise ANI values of these 19 strains and known species were <95%, indicating that they represented 19 novel species. The comparative genomic analysis showed significant differences in gene content between the two groups with a maximum growth temperature (*T*_max_) of ≤ 20°C and a *T*_max_ of >20°C. A comprehensive and robust phylogenetic tree, including 14 known species and 19 novel species, was constructed and showed five phylogenetic branches based on 265 concatenated single-copy gene sequences. The *T*_max_ parameter had a strong phylogenetic signal, indicating that the temperature adaptation of *Cryobacterium* was largely through vertical transfer rather than horizontal gene transfer and was affected by selection. Furthermore, using polyphasic taxonomy combined with phylogenomic analysis, we proposed 19 novel species of the genus *Cryobacterium* by the following 19 names: *Cryobacterium serini* sp. nov., *Cryobacterium lactosi* sp. nov., *Cryobacterium gelidum* sp. nov., *Cryobacterium suzukii* sp. nov., *Cryobacterium fucosi* sp. nov., *Cryobacterium frigoriphilum* sp. nov., *Cryobacterium cryoconiti* sp. nov., *Cryobacterium lyxosi* sp. nov., *Cryobacterium sinapicolor* sp. nov., *Cryobacterium sandaracinum* sp. nov., *Cryobacterium cheniae* sp. nov., *Cryobacterium shii* sp. nov., *Cryobacterium glucosi* sp. nov., *Cryobacterium algoritolerans* sp. nov., *Cryobacterium mannosilyticum* sp. nov., *Cryobacterium adonitolivorans* sp. nov., *Cryobacterium algoricola* sp. nov., *Cryobacterium tagatosivorans* sp. nov., and *Cryobacterium glaciale* sp. nov. Overall, the taxonomy and genomic analysis can improve our knowledge of phenotypic diversity, genetic diversity, and evolutionary characteristics of *Cryobacterium*.

## Introduction

Glaciers represent an important low-temperature ecosystem. The glacier surface encompasses a variety of habitats for cold-adapted microorganisms, including uppermost ice and snow, surface streams, ponds, cryoconite holes, and moraines (Boenigk et al., 2006). These habitats constitute an important ecological area in the glacier, known as the supraglacial ecological zone (Hotaling et al., 2017). Microorganisms living in the supraglacial ecological zone have attracted increasing attention, and many studies have deepened our understanding of their diversity, speciation, and evolution (Liu et al., 2017, 2019b; Nicholes et al., 2019). DNA sequencing technology has provided insights into the composition of the supraglacial microbial community mainly composed of algae, *Cyanobacteria*, fungi, bacteria, viruses, and protozoa (Anesio et al., 2017). Though inhabited by a variety of prokaryotic and eukaryotic microorganisms, the supraglacial community contains predominantly autotrophic and heterotrophic bacteria, which are exposed to oligotrophic, solar radiation, and periodic freezing–thawing effects (Hotaling et al., 2017).

The recent focus has been on unknown extremophilic microorganisms and their metabolic characteristics (Silva et al., 2021). Therefore, obtaining pure cultures of extreme microorganisms for further classification and physiological and evolutionary studies is essential. In the last decade, we isolated thousands of heterotrophic bacterial strains from the surface samples of glaciers in Western China, including some common taxa in the cryosphere, such as *Arthrobacter, Cryobacterium, Flavobacterium, Polaromonas, Sphingomonas*, and *Janthinobacterium* (Liu et al., 2015). Studies investigating these strains have promoted not only the discovery and validation of new species (Liu et al., 2019a, 2020b, 2021b; Zhang et al., 2020; Yang et al., 2021) but also the understanding of novel functions (Liu et al., 2021a) and evolutionary characteristics (Liu et al., 2020a) of cold-adapted bacteria.

The genus *Cryobacterium* represents a group specific to low-temperature environments. It was proposed by Suzuki et al. (1997) with *Cryobacterium psychrophilum* as the type species. It belongs to the class Actinobacteria and the family Microbacteriaceae. Most strains of *Cryobacterium* are cold tolerant, and some of them are psychrophilic, growing at ≤ 20°C (Liu et al., 2020a). At the time of writing this manuscript, *Cryobacterium* included 14 species with validly published names (Parte et al., 2020), of which 12 species were isolated from the cryosphere. All type strains, except *Cryobacterium tepidiphilum* NEAU-85^T^, grew at a temperature of < 10°C (Wang et al., 2019). The maximum growth temperature (Tmax) of nine species was less than 20°C (Suzuki et al., 1997; Reddy et al., 2010; Liu et al., 2012, 2013, 2018a, 2019c, 2020b). Furthermore, the other five species had a *T*_max_ range of 26–30°C and could grow well not only at < 20°C but also at >20°C, indicating a broader temperature tolerance range compared with the psychrophilic species. Because few pure culture strains are available, the investigations related to its phylogeny and biodiversity are comparatively limited. Liu et al. (2020a) conducted an adaptive evolution analysis based on the genomic sequences of 78 *Cryobacterium* strains and found that these strains showed thermotolerance divergence with phylogenetic cohesion, which was closely related to the GC3 content. These findings indicated that *Cryobacterium* was a valuable bacterial genus for studying adaptive evolution under temperature stress. Therefore, it is important to isolate additional *Cryobacterium* strains from the cryosphere and identify novel species.

Our previous studies showed that the pure cultures of *Cryobacterium*, including strains isolated in our laboratory and type strains of known species, could be classified into at least more than 30 species with high genetic diversity (Liu et al., 2020a). In this study, we analyzed more than 100 genome sequences of *Cryobacterium* strains in GenBank. We inferred that at least 44 species of the genus *Cryobacterium* existed with pure cultures. Therefore, many novel species remain to be classified, identified, and validly published. We described 19 strains, which we isolated from glaciers, and proposed 19 novel species using a polyphasic approach. Furthermore, we divided 79 *Cryobacterium* strains with available *T*_max_ traits into two groups based on their ability to grow at a temperature of more than 20°C and conducted a genome analysis. This study provided new insights into the cold-adaptation mechanisms and ecological diversification of the genus *Cryobacterium*.

## Materials and methods

### Genome information, annotation, and ANI calculation

The whole-genome sequences of the genus *Cryobacterium* were downloaded from the National Center for Biotechnology Information (NCBI) genome database using the BioSAK program (https://github.com/songweizhi/BioSAK). The genomes were classified using the Genome Taxonomy Database Toolkit (GTDB-Tk) v.2.1.0 (Chaumeil et al., 2019) based on the GTDB Release R207_v2 database (Parks et al., 2022). The CheckM v.1.1.3 program (Parks et al., 2015) was used to check the completeness and contamination values of the genomes. The average nucleotide identity (ANI) values were calculated using the FastANI program (Jain et al., 2018). The symmetric pairwise ANI dissimilarity matrix was clustered using the average linkage hierarchical clustering method with the “bactaxR” package (Carroll et al., 2020) in R 4.0.3. The genomes were annotated using Prokka software (Seemann, 2014). The protein sequence was also functionally annotated and classified into Cluster of Orthologous Groups (COG) categories using the BLAST+ program (Camacho et al., 2009) based on the latest COG database (Galperin et al., 2021). Carbohydrate-active enzymes (CAZymes) were identified using the CAZy database V11 (Drula et al., 2022). The growth temperature data were obtained from the studies by Wang et al. (2019), Gong et al. (2020), and Liu et al. (2020a). The strains with available *T*_max_ were divided into two groups: strictly psychrophilic (SP, *T*_max_ ≤ 20°C) and psychrotolerant (PT, *T*_max_ > 20°C).

### Strains and culture conditions

In total, 19 *Cryobacterium* strains, which were selected for polyphasic taxonomic analysis, were isolated from ice, cryoconite, and meltwater samples in the supraglacial zone of Xinjiang No. 1, Toumingmengke, Hailuogou, and Midui glaciers of China, as listed in Table 1 and described by Liu et al. (2020a). All strains were cultivated in a peptone, yeast extract, and glucose (PYG) medium (Liu et al., 2012) and routinely incubated at 15°C.

**Table 1:** Nineteen novel *Cryobacterium* species proposed in this study.

**Strain**	**Proposal name**	**CGMCC No**.	**NBRC No**.	**Isolation source**	**16S rDNA accession no**.	**Genome accession no**.
Sr54^T^	*Cryobacterium serini*	1.9249	114034	Ice	JX949302	GCA_004404025.1
Sr59^T^	*Cryobacterium lactosi*	1.9254	114035	Ice	JX949303	GCA_004403945.1
Hz16^T^	*Cryobacterium gelidum*	1.9272	114048	Ice	JX949290	GCA_004403985.1
Sr39^T^	*Cryobacterium suzukii*	1.9276	114032	Ice	JX949299	GCA_004403185.1
Hh4^T^	*Cryobacterium fucosi*	1.9290	114036	Ice	JX949272	GCA_004403515.1
Hh14^T^	*Cryobacterium frigoriphilum*	1.9297	114037	Ice	JX949277	GCA_004403305.1
TMT1-51^T^	*Cryobacterium cryoconiti*	1.9350	114038	Cryoconite	JX949903	GCA_004403065.1
TMT1-1^T^	*Cryobacterium lyxosi*	1.9465	113798	Cryoconite	JX949920	GCA_004403345.1
TMT1-23-1^T^	*Cryobacterium sinapicolor*	1.9483	113799	Cryoconite	JX949927	GCA_004403465.1
TMT2-16^T^	*Cryobacterium sandaracinum*	1.9503	114039	Cryoconite	JX949884	GCA_004403415.1
TMT2-48-2^T^	*Cryobacterium cheniae*	1.9517	114040	Cryoconite	JX949892	GCA_004403395.1
TMT1-22^T^	*Cryobacterium shii*	1.9687	114041	Cryoconite	JX949935	GCA_004402595.1
MDB1-5^T^	*Cryobacterium glucosi*	1.9741	114042	Ice	JX949731	GCA_004402235.1
MDT1-3^T^	*Cryobacterium algoritolerans*	1.9782	114043	Cryoconite	JX949739	GCA_004402785.1
RHLT2-21^T^	*Cryobacterium mannosilyticum*	1.10060	114044	Cryoconite	JX949475	GCA_004402705.1
RHLS22-1^T^	*Cryobacterium adonitolivorans*	1.10101	114045	Melt water	JX949476	GCA_004402695.1
MDB2-B^T^	*Cryobacterium algoricola*	1.11135	114047	Ice	JX949747	GCA_004402485.1
Sr47^T^	*Cryobacterium tagatosivorans*	1.11221	114033	Ice	JX949300	GCA_004402215.1
HLT2-23^T^	*Cryobacterium glaciale*	1.11085	114046	Ice	JX949477	GCA_004402535.1

### Phylogenetic and phylogenomic analyses

The 16S rRNA gene sequences of the aforementioned strains were retrieved from GenBank using accession numbers (Table 1; Liu et al., 2020a) and compared with the latest GenBank database using BLAST+ software (Camacho et al., 2009) to search their close phylogenetic neighbors. The complete 16S rRNA gene sequences were also retrieved from the genome sequences using RNAmmer (Lagesen et al., 2007) for phylogenetic analysis. After multiple sequence alignment using ClustalW (Thompson et al., 1994), the neighbor-joining tree was built with 1,000 bootstrap replicates using the Molecular Evolutionary Genetics Analysis V.5.2 software (Tamura et al., 2011). Kimura's two-parameter model was used to calculate the genetic distances (Kimura, 1980).

Single-copy core genes were extracted from the genome sequences using the GET_HOMOLOGUES program (Contreras-Moreira and Vinuesa, 2013). The phylogenetic markers were selected using the GET_PHYLOMARKERS program for constructing species trees (Vinuesa et al., 2018). The concatenated sequences of the top-ranking phylogenetic marker genes were aligned using Clustal Omega software (Sievers and Higgins, 2014). The phylogenomic tree was constructed using the maximum-likelihood algorithm with a GTR+F+I+G4 model in the IQ-TREE software (Nguyen et al., 2015) based on the concatenated gene sequences with 1,000 bootstrap replicates. Blomberg's *K* (Blomberg et al., 2003) was measured based on 1,000 randomizations using the “phytools” package in R 4.0.3 to test the phylogenetic signal of the *T*_max_ trait. Blomberg's *K* values were tested for the null hypothesis that the trait values were randomly distributed in the phylogenetic tree.

### Physiology and chemotaxonomy

The 19 *Cryobacterium* strains were subjected to polyphasic taxonomic analysis. The morphology of the colonies was determined after culturing on PYG agar for 7 days. The cellular morphology was examined *via* transmission electron microscopy using a JEM-1400 transmission electron microscope (JEOL Ltd., Tokyo, Japan). The growth at various NaCl concentrations [0–4.0% (*w*/*v*) at 0.5% intervals] and pH levels (ranging from pH 5.0 to 10.0 at intervals of 1 pH unit) were tested in the presence of PYG broth for 10 days. The hydrolysis of casein, starch, and Tween 80 was performed according to Smibert and Krieg (1994). The agar plates for the starch hydrolysis test were flooded with iodine solution after growth. The colorless areas around the colonies were observed for determining casein and starch hydrolysis. Lipolysis was tested on PYG agar with 1% (*v*/*v*) Tween 80 by observing the formation of an oily iridescent sheen and its vicinity on the surface of the medium. The use of a sole carbon source was tested with a GEN III MicroStation (Biolog). The enzyme activities and other biochemical tests were performed using the API 20E, 20NE, and ZYM strips (bioMérieux; Marcy-l'Étoile, France) following the manufacturer's protocols.

For analyzing the cellular fatty acid composition, the cells of the tested strains were harvested from colonies on the same sectors of the PYG plates after incubation at 15°C. The saponified and methylated fatty acids were extracted according to the MIDI 6.0 protocol (Sasser, 1990). The samples were analyzed using an Agilent 6890N gas chromatography system (Agilent Technologies, CA, USA) equipped with an HP-ULTRA two-capillary column (25 m × 0.2 mm × 0.33 μm; Agilent) and a flame ionization detector (Agilent) and identified using the TSBA6 database of the Microbial Identification System (Sherlock version 6.0B). The cells were harvested from PYG broth after incubation at 15°C for 4 days and freeze-dried for analyzing menaquinones and polar lipids. Menaquinones were extracted and purified based on the method of Collins (1985) and analyzed by HPLC using a YMC-Pack ODS-A column (150 × 4.6 mm^2^) with the elution of methanol/isopropyl ether (3:1, *v*/*v*) at a flow rate of 1 ml/min, followed by the detection of ultraviolet absorbance at a wavelength of 275 nm. Polar lipids were extracted and separated on silica gel 60 plates (Merck 1.05553) *via* two-dimensional thin-layer chromatography (Komagata and Suzuki, 1988).

## Results

### Diversity of *Cryobacterium*

**Figure 1:**
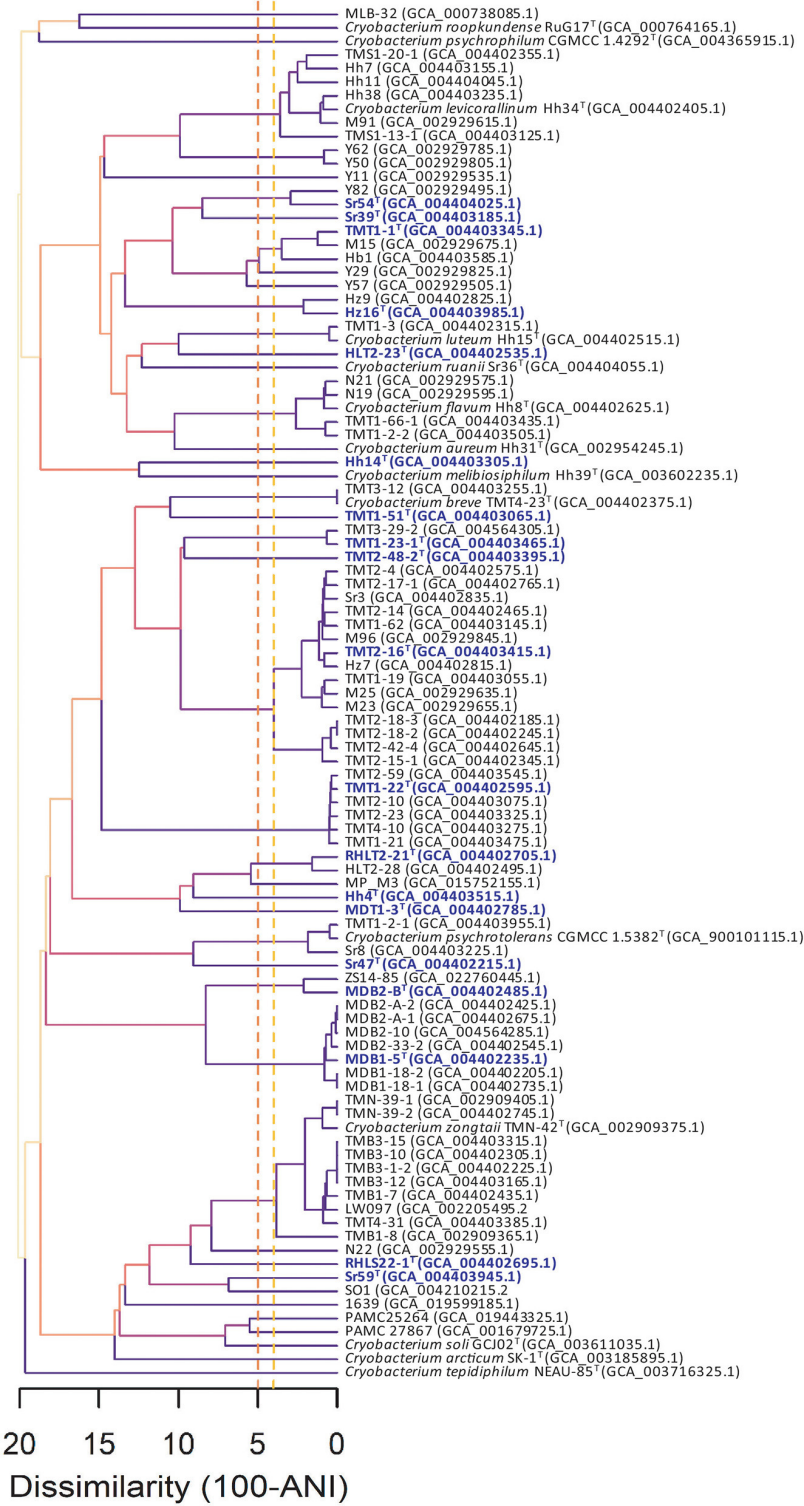
Cluster analysis based on pairwise ANI values of 101 *Cryobacterium* strains. Accession numbers of the genomic sequences are given in parentheses. Nineteen strains representing the novel species described in this study are in purple color.

At present, 120 genome sequences are temporarily classified into the genus *Cryobacterium* in GenBank. After removing duplicate strains and metagenome assembly samples, 103 genomes were downloaded using the BioSAK tool (Supplementary Table 1). The completeness of all the genome sequences was above 96%, of which, the completeness of 102 genomes was above 98%. Based on the GTDB Release R207_v2 database, 101 strains were classified into the genus *Cryobacterium*, and the other two strains BB307 and BB736 were classified into the genus *Terrimesophilobacter* (Supplementary Table 1). The 101 strains could be classified into 44 species according to the threshold for species delineation of 96% (Richter and Rosselló-Móra, 2009) and the FigANI value cluster analysis (Figure 1). It indicates that at least 44 species of *Cryobacterium* have been isolated and cultured to date. As only 14 species with validly published names exist at present, at least 30 new species need to be classified and described. In addition, the cluster diagram of ANI values showed higher phylogenetic diversity of the genus *Cryobacterium* than in a previous study by Liu et al. (2020a); some species showed intraspecific diversity. For example, one unknown species contained 15 strains; *Cryobacterium zongtaii* contained 11 strains, which were clustered into three branches; and *Cryobacterium levitaulinum* contained seven strains and was divided into three branches (Figure 1).

### Comparative genomic analysis

**Figure 2:**
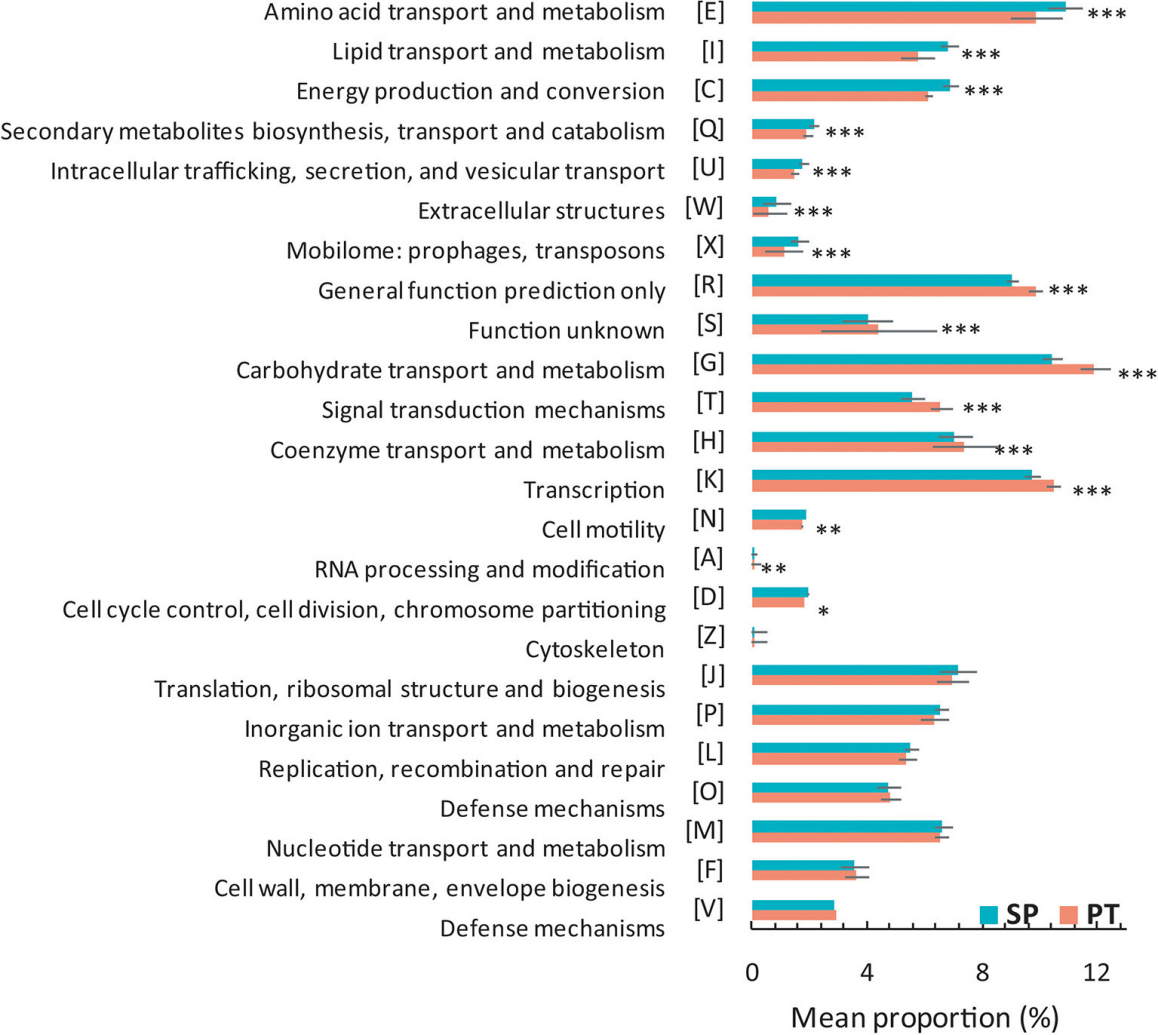
Proportional differences of COG category between the SP and PT groups. Two groups were defined by the strains with *T*_max_ ≤ 20°C (SP) and *T*_max_ > 20°C (PT). *P*-values were obtained using the Student's *t-*test. ^***^P < 0.005; ^**^P = 0.005–0.01; ^*^P = 0.01–0.05.

Among the 101 strains, the *T*_max_ trait of 79 strains was available. These strains were divided into SP and PT groups for comparative genomic analysis based on *T*_max_ (Supplementary Table 1). The gene content showed differences between the two groups (Figure 2). Compared with the PT group, the proportions in the SP group related to “amino acid transport and metabolism,” “lipid transport and metabolism,” “energy production and conversion,” “secondary metabolite biosynthesis, transport, and catabolism,” “intracellular trafficking, secretion, and vesicular transport,” “extracellular structures,” “mobilome: prophages and transposons,” “cell motility,” and “cell cycle control, cell division, and chromosome partitioning” were significantly higher (*P* < 0.05); however, the proportions of “carbohydrate transport and metabolism,” “general function prediction only,” “signal transduction mechanisms,” “coenzyme transport and metabolism,” “transcription,” and “function unknown” were significantly lower (*P* < 0.05).

**Figure 3:**
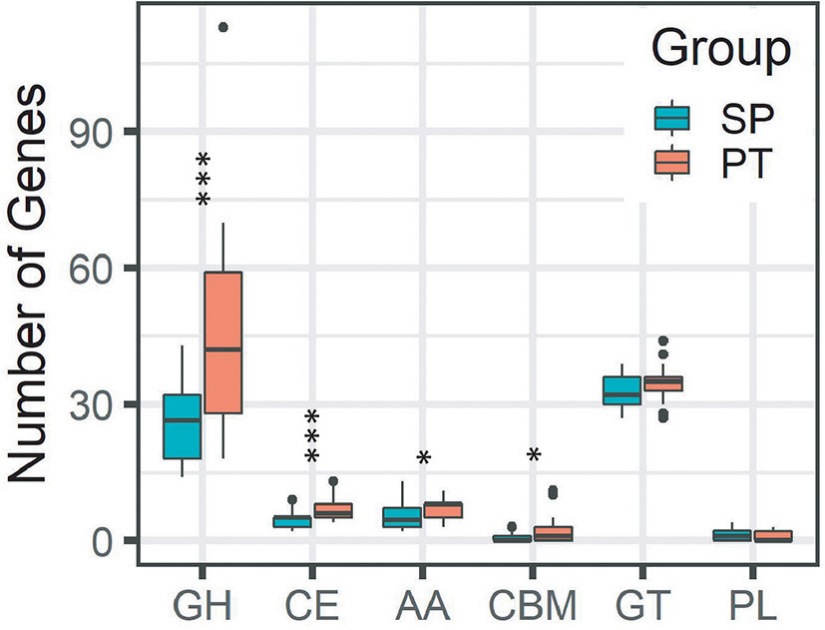
Differences in the predicted CAZyme profiles between the SP and PT groups. Two groups were defined by the strains with *T*_max_ ≤ 20°C (SP) and *T*_max_ > 20°C (PT). ^***^P < 0.005; ^*^P = 0.01–0.05. Enzyme classes: (GH), glycoside hydrolases; (CE), carbohydrate esterases; (AA), auxiliary activities; (CBM),carbohydrate-binding modules; glycosyltransferases, and (PL), polysaccharide lyases.

We compared the numbers of genes of CAZyme families using the CAZy database to estimate the ability to assemble, modify, and break down carbohydrates and glycoconjugates between SP and PT groups. The differences in the predicted CAZyme profiles were found between SP and PT groups (Figure 3). The number of enzymes involved in glycoside hydrolase (GH) and carbohydrate esterase (CE) was significantly lower in the SP group compared with the PT group (*P* < 0.005); meanwhile, the number of enzymes involved in auxiliary activities (AAs) and carbohydrate-binding modules (CBMs) was also significantly lower (*P* < 0.05).

### Phylogenetic and phylogenomic analyses

Among the 30 new species found in this study, 19 were isolated from the glacier samples in Western China. Furthermore, 19 *Cryobacterium* strains were selected as type strains and deposited into two publicly accessible service collections for further phylogenetic and phylogenomic studies (Table 1). The symmetric pairwise ANI values between the 19 new type strains and 14 type strains of the known species ranged from 78.58 to 91.76%. Therefore, they represented 19 novel species belonging to the genus *Cryobacterium*.

The complete 16S rRNA gene sequences of the 33 type strains were also retrieved from the genome sequences. All *Cryobacterium* strains contained one copy of the 16S rRNA gene, expect for *Cryobacterium arcticum* SK-1^T^, which contained three copies. The phylogenetic analysis based on 16S rRNA gene sequences showed that all of the 19 new type strains were members of the genus *Cryobacterium* (Supplementary Figure 1). The sequence similarities of these strains with their respective closest type strains ranged from 98.76 to 100% (Supplementary Table 2). Obviously, it was difficult to differentiate them from their closely related strains using the 16S rRNA gene sequence, as they shared high levels of similarity, exceeding the 98.65% cutoff for species distinction suggested by Kim et al. (2014).

**Figure 4:**
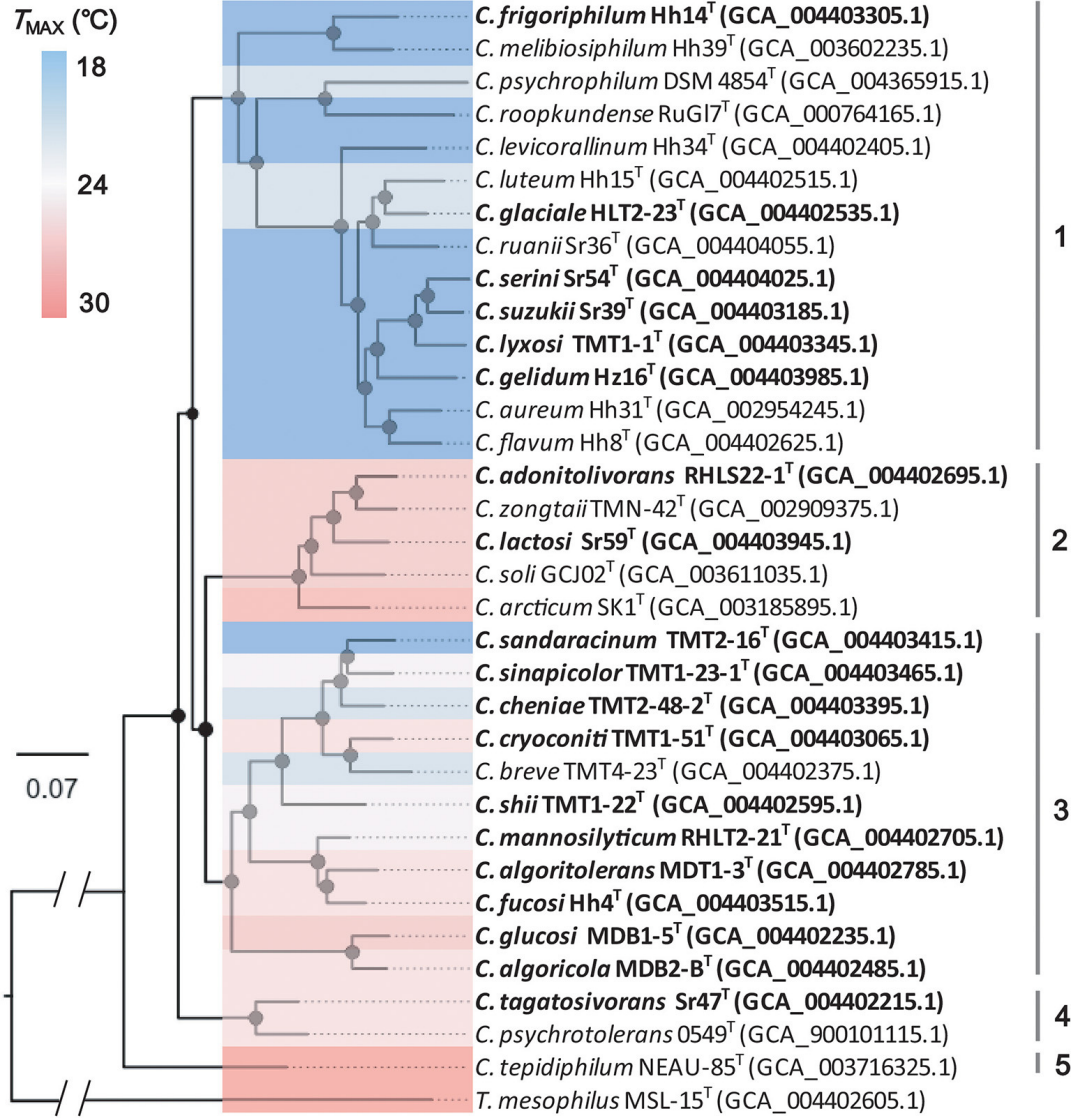
Phylogeny of the genus *Cryobacterium* using the maximum-likelihood algorithm based on the 265 concatenated single-copy gene sequences. Accession numbers of the genomic sequences are given in parentheses. Bootstrap values >70% are marked as black dots on the internal nodes. The bar represents 0.07 nt substitutions per site.

A phylogenomic analysis of the aforementioned strains was carried out to determine their phylogenetic relationship. Furthermore, 479 single-copy core genes were extracted and 265 top-ranking phylogenetic markers were selected from the genomes. Following sequence alignment, the concatenated sequences of phylogenetic markers were used to construct the species tree using the maximum-likelihood method. A robust tree with strong bootstrap support was obtained (Figure 4). The 33 strains of *Cryobacterium* were clearly separated from each other and grouped into five clusters based on phylogenetic similarity.

Cluster 1 consisted of 14 strains, including Hh14^T^, HLT2-23^T^, Sr54^T^, Sr39^T^, TMT1-1^T^, Hz16^T^, and eight type strains of known species with validly published names: *Cryobacterium melibiosiphilum* Hh39^T^, *Cryobacterium roopkundense* RuGl7^T^, *Cryobacterium psychrophilum* DSM 4854^T^, *Cryobacterium levicorallinum* Hh34^T^, *Cryobacterium luteum* Hh15^T^, *Cryobacterium ruanii* Sr36^T^, *Cryobacterium aureum* Hh31^T^, and *Cryobacterium flavum* Hh8^T^. In total, 11 of these strains were isolated from Xinjiang's No. 1 glacier in China. Strains RHLS22-1^T^, Sr59^T^, *Cryobacterium zongtaii* TMN-42T^T^, *Cryobacterium soli* GCJ02^T^, and *C. arcticum* SK-1^T^ formed cluster 2. Cluster 3 comprised 11 strains, including TMT2-16^T^, TMT1-23-1^T^, TMT2-48-2^T^, TMT1-51^T^, TMT1-22^T^, RHLT2-21^T^, MDT1-3^T^, Hh4^T^, MDB1-5^T^, MDB2-B^T^, and *Cryobacterium breve* TMT4-23^T^. Strains Sr47^T^ and *Cryobacterium psychrotolerans* 0549^T^ clustered together and formed cluster 4. Strain *C. tepidiphilum* NEAU-85^T^ was separated from other clusters and formed a distinct and independent branch. The species tree based on the 265 single-copy core genes yielded a comprehensive phylogenetic structure of the genus *Cryobacterium*. All strains in the species tree represented different species, which greatly expanded the species diversity of the genus *Cryobacterium*. Therefore, we proposed 19 novel species under the genus *Cryobacterium* based on pairwise ANI values and phylogenetic analysis. The proposed names are listed in Table 1.

The *T*_max_ trait of *Cryobacterium* species showed large differences between different phylogenetic branches (Figure 4). The psychrophilic species were mainly distributed in cluster 1. We used Blomberg's *K* to measure the strength of the signal relative to the Brownian motion (BM) model of the *T*_max_ trait to test the evolution of *Cryobacterium* to temperature. For the *T*_max_ trait, the closely related species tended to exhibit similar phylogenetic signals (Blomberg's *K* = 3.10; *P*-value = 0.001). The value of Blomberg's *K* > 1 indicated that the similarity between closely related species was higher than expected under the BM model (Blomberg et al., 2003), showing the phylogenetic conservation of the *T*_max_ trait and implying that selection played a role in the *T*_max_ trait evolution. This result was consistent with our previous finding related to the adaptive evolution of *Cryobacterium* strains (Liu et al., 2020a).

### Phenotypic and chemotaxonomic characteristics

The phenotypic and chemotaxonomic characteristics of 19 novel species of the genus *Cryobacterium* were evaluated and showed a phenotypic diversity (Supplementary Tables 3, 4). These strains formed colorful colonies and were found to be Gram-positive, rod-shaped, and catalase-positive bacteria. Cells of 15 strains carried a single flagellum, while no flagellum was detected in the cells of the other four strains (Supplementary Figure 2). They showed growth in a pH range of 6.0/7.0–9.0/10.0, varying with different strains. They could all grow at 0% NaCl concentration, while the maximum NaCl tolerance concentration varied greatly from 1 to 5% (Supplementary Table 3). Several differences were observed in their ability to use a sole carbon source (Supplementary Table 4). The number of carbon sources that these strains could use showed a correlation with their *T*_max_ (*R*^2^ = 0.69 and *P* = 0.001). The strains with higher *T*_max_ could use more carbon sources.

The cellular fatty acid composition of novel strains is shown in Table 2. All strains contained anteiso-C15:0 as the predominant fatty acids, which was consistent with the other members of the genus Cryobacterium (Liu et al., 2018b). Different strains carried different secondary dominant components, such as *iso*-C_15:0_, *iso*-C_16:0_, *anteiso*-C_17:0_, and *iso*-C_17:1_ ω5*c*. Their polar lipid profile included diphosphatidylglycerol, phosphatidylglycerol, one unidentified glycolipid, and one unidentified lipid, which was consistent with the description of the genus Cryobacterium (Liu et al., 2018b). All strains contained multiple menaquinone components. The dominant components of most strains included MK-10 and/or MK-9, and MK-11. However, strains MDT1-3^T^, RHLT2-21^T^, and Sr47^T^ contained MK-11 and MK-12 as the primary menaquinones with minor amounts of MK-10. Other detailed phenotypic characteristics of these strains are listed in the species description.

**Table 2:** Cellular fatty acid composition (%) of the type strains of 19 novel *Cryobacterium* species.

**Strain**	***Iso*-C_15:1_**	***Anteiso*-C_15:1_**	***Iso*-C_17:1_ ω5*c***	***Iso*-C_14:0_**	***Iso*-C_15:0_**	***Iso*-C_16:0_**	***Iso*-C_17:0_**	***Anteiso*-C_15:0_**	***Anteiso*-C_17:0_**	**C_16:0_**
Sr54^T^	Tr	7.1	Tr	1.1	13.2	16.2	1.8	47.3	10.3	1.7
Sr59^T^	4.7	17.7	–	Tr	Tr	24.1	Tr	31.9	14.9	3.5
Hz16^T^	Tr	8.0	Tr	1.4	8.7	23.5	2.1	40.4	10.1	4.5
Sr39^T^	Tr	9.0	Tr	Tr	5.4	15.6	1.2	47.3	18.7	1.4
Hh4^T^	4.6	11.8	–	Tr	Tr	16.1	Tr	52.2	10.0	2.6
Hh14^T^	2.8	11.3	Tr	1.6	12.4	23.2	2.2	30.4	11.4	3.3
TMT1-51^T^	10.9	14.5	–	Tr	Tr	17.5	Tr	25.7	11.6	15.1
TMT1-1^T^	Tr	8.5	Tr	Tr	1.4	19.8	Tr	45.2	19.4	3.8
TMT1-23-1^T^	Tr	6.8	16.8	Tr	Tr	19.4	–	25.0	11.2	6.0
TMT2-16^T^	7.5	9.8	8.3	Tr	Tr	22.2	Tr	30.3	12.8	6.4
TMT2-48-2^T^	14.3	11.8	–	Tr	Tr	19.2	Tr	29.3	12.5	9.1
TMT1-22^T^	8.5	12.3	–	Tr	1.2	19.0	Tr	37.0	11.1	7.6
MDB1-5^T^	1.2	4.2	1.0	Tr	1.1	10.1	Tr	62.1	16.4	2.9
MDT1-3^T^	5.4	9.3	–	Tr	Tr	24.1	Tr	38.0	14.2	5.2
RHLT2-21^T^	4.7	5.2	4.5	Tr	2.7	18.6	Tr	43.1	12.1	7.0
RHLS22-1^T^	4.5	8.2	8.6	Tr	Tr	20.6	Tr	32.3	18.2	5.3
MDB2-B^T^	2.1	9.2	–	1.3	1.5	18.4	Tr	56.0	6.8	3.6
Sr47^T^	2.3	17.7	–	Tr	14.6	14.4	1.6	34.3	10.4	2.6
HLT2-23^T^	Tr	8.3	Tr	1.4	8.7	23.9	1.4	43.0	9.6	2.3

Values are percentages of the total fatty acids. tr, traces (< 1% of the total fatty acids); –, not detected. The results were all from this study.

## Discussion

Temperature is a crucial ecological factor for microorganisms. Microorganisms have a specific range of temperature tolerance and are constantly affected by changes in the external ambient temperature. A continental-scale study of *Cyanobacteria* found that temperature was the main factor affecting cyanobacterial populations, and differences were observed in temperature tolerance among populations at different latitudes (Garcia-Pichel et al., 2013). The *Spumella* morphospecies in different climatic regions showed adaptation to different temperatures, and the temperatures for maximum and optimum growth were significantly correlated with habitat temperature (Boenigk et al., 2006, 2007). Based on the analysis of whole-genome single-nucleotide polymorphisms and chromosomal regions, Ellison et al. (2011) found that temperature at different latitudes led to the genomic divergence of two *Neurospora crassa* populations and differences in their temperature fitness. Therefore, temperature plays an important role not only in the structure and composition of microbial communities but also in genome variation and evolution.

*Cryobacterium* is a typical cold-active genus inhabiting the cryosphere. Taxonomic and comparative genomic studies could provide new insights into its adaptive evolution. In this study, we compared the gene content of two groups containing 79 *Cryobacterium* strains. Although no significant difference was observed in the genome size of these strains in the two groups, several differences in the gene content were observed between the groups. The higher number of genes involved in several COG categories (i.e., “amino acid transport and metabolism,” “lipid transport and metabolism,” “secondary metabolite biosynthesis, transport, and catabolism,” “intracellular trafficking, secretion, and vesicular transport,” “extracellular structures,” “mobilome: prophages and transposons,” “cell motility,” and “cell cycle control, cell division, and chromosome partitioning”) was found in the SP group than in the PT group, indicating that the acquisition of genes belonging to these functions might be related to their *T*_max_ trait. Some genes (i.e., “general function prediction only” and “function unknown”) were less abundant in the SP group than in the PT group, which might not be necessary for survival. The loss of some nonessential genes in the SP group might help them save energy in a cold environment. As reported by Liu et al. (2020), gene gain/loss events occurred frequently in the genome of *Cryobacterium*. These two evolutionary processes might be proactive processes leading to the niche differentiation of *Cryobacterium*. Interestingly, the number of genes related to carbohydrate transport and metabolism was significantly lower in the SP group than in the PT group (Figure 2). Carbohydrate metabolism is central to whole biological metabolism, mainly including synthesis, decomposition, modification, and other processes, which require enzymes with different functions. The CAZymes classified into families GH and CE were involved in carbohydrate degradation (Wardman et al., 2022). The amounts of GH and CE in the SP group were significantly lower (Figure 3), suggesting that the ability of the SP group to metabolize carbohydrates might be reduced. This finding was consistent with the results of phenotypic experiments, which showed that the strains with lower *T*_max_ could use fewer carbon sources. However, whether this difference in carbon source utilization capacity has a causal relationship with the *T*_max_ remains to be further explored.

In this study, we also inferred that the genus *Cryobacterium* contains at least 44 species based on genome sequences of pure cultures in the NCBI database, including 19 species isolated from glaciers in Western China. We proposed them as 19 novel species using the polyphasic taxonomic method combined with phylogenomic analysis. Therefore, the number of species with validly published names under this genus was expanded from 14 to 33, which significantly increased our understanding of the species diversity of *Cryobacterium*. The species in the genus *Cryobacterium* showed phenotypic diversity, such as the difference in their maximum NaCl tolerance concentrations. The salt concentration in the microenvironment changed during the freeze–thaw cycles in the supraglacial zone, resulting in variations in osmotic pressure. *Cryobacterium* achieved the adaptation ability at different NaCl concentrations perhaps for this reason. The *T*_max_ of *Cryobacterium* species also showed large-scale differences among different species. All species collected from the cryosphere (32 species) showed strong cold adaptability (growing at 0°C), which included 16 species with a *T*_max_ of ≤ 20°C and other species that failed to grow at a temperature of more than 26°C. Blomberg's *K* values were tested for the null hypothesis of the absence of the phylogenetic signal, which fit the model of neutral evolution. According to Blomberg et al. (2003), *K* values >1 indicated that the related species resembled each other more than expected under BM, implying that the *T*_max_ trait was phylogenetic conservation and affected by selection rather than by neutral drift. The codon usage bias (Liu et al., 2020a) and gene content changed under low-temperature selection pressure, resulting in the diversification of species and differences in temperature adaptability. Therefore, we inferred that under the selective pressure of low-temperature conditions in the cryosphere, *Cryobacterium* acquired strong adaptability to low temperatures but lost the ability to survive in the external environment at higher temperatures. The dispersal barrier was finally formed due to the ecological differentiation between the cryosphere and the external environment, resulting in *Cryobacterium* as a specific group in the cryosphere. Hence, *Cryobacterium* occupied specific ecological niches and formed several novel and distinct species with diverse phenotypes in long-term cold habitats.

Most psychrophilic species were present in cluster 1 of the species tree of the genus *Cryobacterium*, while the other three appeared in cluster 3. The *Cryobacterium* species with different *T*_max_ values could be found in the same glacier sample since both the SP and PT groups were well adapted at low temperatures. Therefore, the genome-wide selective sweep (Polz et al., 2013) was less likely to occur in the case of *Cryobacterium*. However, the low *r*/*m* and ρ/θ values within the genus *Cryobacterium* calculated by Liu et al. (2020a) indicated a low rate of interspecies recombination, suggesting that gene-specific sweeps (Cohan and Koeppel, 2008) also could not explain the cold-adapted evolution of *Cryobacterium*. The phylogenetic conservation of the *T*_max_ trait implied that the temperature adaptation was mainly through vertical transfer rather than horizontal transfer. Previous studies reported that the adaptation of bacteria to temperature changes involved multiple genes, such as the genes related to the fluidity of the cellular membrane, osmotic and oxidative stress, polysaccharide transporter, ice-binding protein, cold-shock protein, and carotenoid biosynthesis (Casanueva et al., 2010). Therefore, as a multigene trait, temperature adaptation may not be easily acquired through horizontal gene transfer. Hence, we inferred that the transmission of temperature adaptation of *Cryobacterium* was largely *via* vertical transfer, resulting in the formation of phylogenetic clustering of temperature adaptation.

## Conclusion

In this study, 19 *Cryobacterium* strains originally isolated from the glaciers in China were classified into 19 novel species using polyphasic taxonomy combined with phylogenomic analysis. Based on 265 single-copy core genes, a comprehensive and robust phylogenetic tree including 33 species belonging to the genus *Cryobacterium* was constructed with five phylogenetic branches. A comparative genomics analysis of the genus *Cryobacterium* was performed, indicating significant differences in the gene content between the groups with different *T*_max_ values (≤ 20°C and >20°C). Furthermore, a strong phylogenetic signal of the *T*_max_ trait was detected, suggesting that the temperature adaptation of *Cryobacterium* was largely *via* vertical transfer rather than *via* horizontal transfer and was affected by selection. Hence, the proposal and description of 19 novel species and the comparative genomic analysis enhanced our understanding of the phenotype and genetic diversity of the genus *Cryobacterium* and also provided some insights into the ecosystem of the supraglacial zone in glaciers.

### Description of *Cryobacterium serini* sp. nov.

*Cryobacterium serini* (se.ri'ni. N.L. gen. n. *serini*, of serine, referring to the utilization of D-serine).

Cells are Gram-positive, aerobic, non-motile, rod-shaped, and 1.5–1.7 × 0.7–0.8 μm in size. Cells do not carry any flagella. Colonies are gold-colored, convex, round, and 1.0 mm in diameter after 7 days of incubation on PYG plates at 14°C. Growth occurs at 0–18°C, pH 6.0–10.0, and in the presence of 0–3.0% (w/v) NaCl. The optimum growth temperature is 10–14°C. Positive for catalase, but negative for oxidase. Reduces nitrate to nitrite. Does not hydrolyze Tween 80, gelatin, casein, and starch. Hydrolyze esculine. Indole and H_2_S are not formed. Positive for alkaline phosphatase, leucine arylamidase, citrate utilization, Voges–Proskauer test, and β-galactosidase. Utilizes the following substances as carbon sources: D-maltose, D-trehalose, D-cellobiose, sucrose, stachyose, α-D-glucose, D-mannose, D-galactose, D-sorbitol, D-arabitol, glycerol, D-serine, gelatin, glycyl-L-proline, L-alanine, L-arginine, L-serine, pectin, D-galacturonic acid, D-gluconic acid, mucic acid, quinic acid, D-saccharic acid, L-lactic acid, citric acid, D-malic acid, L-malic acid, Tween 40, γ-aminobutyric acid, α-hydroxybutyric acid, β-hydroxy-D,L-butyric acid, acetoacetic acid, and acetic acid. Acids are produced from glycerol, fructose, esculine, starch, and D-arabitol. The major fatty acids are *anteiso*-C_15:0_, *iso*-C_16:0_, *iso*-C_15:0_, *anteiso*-C_17:0_, and *anteiso*-C_15:1_. The predominant menaquinones include MK-10 and MK-9, with minor amounts of MK-8. The polar lipids are diphosphatidylglycerol, phosphatidylglycerol, one unidentified glycolipid, and one unidentified lipid. The DNA G+C content of the type strain is 63.79 %.

The type strain, Sr54^T^ (=CGMCC 1.9249^T^ = NBRC 114034^T^), was isolated from the ice sample obtained from the supraglacial zone of No. 1 glacier in Xinjiang Uygur Autonomous Region, China. The NCBI accession numbers for the 16S rRNA gene and genome sequences are JX949302 and GCA_004404025.1, respectively.

### Description of *Cryobacterium lactosi* sp. nov.

*Cryobacterium lactosi* (lac.to'si. N.L. gen. n. *lactosi*, of lactose).

Cells are Gram-positive, aerobic, motile with a single flagellum, rod-shaped, and 1.3–1.9 × 0.7–0.8 μm in size. Colonies are citrine-colored, convex, round, and 1.0 mm in diameter after 7 days of incubation on PYG plates at 14°C. Growth occurs at 0–26°C, pH 6.0–10.0, and in the presence of 0–5.0% (w/v) NaCl. The optimum growth temperature is 14–20°C. Positive for catalase, but negative for oxidase. Does not reduce nitrate to nitrite. Does not hydrolyze Tween 80, gelatin, casein, and starch. Hydrolyze esculine. Indole and H_2_S are not formed. Positive for leucine arylamidase, β-galactosidase, α-glucosidase, β-glucosidase, and Voges–Proskauer test. Utilizes the following substances as carbon sources: dextrin, D-maltose, D-trehalose, D-cellobiose, gentiobiose, sucrose, D-turanose, stachyose, D-raffinose, α-D-lactose, D-melibiose, β-methyl-D-glucoside, D-salicin, N-acetyl-D-glucosamine, N-acetyl-β-D-mannosamine, N-acetyl-D-galactosamine, N-acetyl neuraminic acid, α-D-glucose, D-mannose, D-fructose, D-galactose, D-fucose, L-rhamnose, inosine, D-sorbitol, D-mannitol, glycerol, glycyl-L-proline, L-alanine, L-aspartic acid, L-glutamic acid, L-histidine, pectin, D-galacturonic acid, L-galactonic acid lactone, D-gluconic acid, D-glucuronic acid, methyl pyruvate, L-lactic acid, citric acid, D-malic acid, L-malic acid, bromosuccinic acid, Tween 40, α-hydroxybutyric acid, β-hydroxy-D,L-butyric acid, α-keto-butyric acid, propionic acid, acetic acid, and formic acid. Acids are produced from glycerol, L-arabinose, D-ribose, D-xylose, galactose, glucose, fructose, mannose, mannitol, methyl-α-D-glucopyranoside, N-acetylglucosamine, esculine, D-cellobiose, D-maltose, D-lactose, D-melibiose, D-saccharose, D-trehalose, D-melezitose, D-raffinose, D-fucose, and L-arabitol. The major fatty acids are *anteiso*-C_15:0_, *iso*-C_16:0_, *anteiso*-C_15:1_, and *anteiso*-C_17:0_. The predominant menaquinone includes MK-10, with minor amounts of MK-9 and MK-11. The polar lipids are diphosphatidylglycerol, phosphatidylglycerol, one unidentified glycolipid, and one unidentified lipid. The DNA G+C content of the type strain is 67.55%.

The type strain, Sr59^T^ (=CGMCC 1.9254^T^ = NBRC 114035^T^), was isolated from the ice sample obtained from the supraglacial zone of No. 1 glacier in Xinjiang Uygur Autonomous Region, China. The NCBI accession numbers for the 16S rRNA gene and genome sequences are JX949303 and GCA_004403945.1, respectively.

### Description of *Cryobacterium gelidum* sp. nov.

*Cryobacterium gelidum* (ge'li.dum. L. neut. adj. gelidum, icy cold, very cold).

Cells are Gram-positive, aerobic, motile with a single flagellum, rod-shaped, and 1.1–1.7 × 0.7–0.9 μm in size. Colonies are golden poppy-colored, convex, round, and 2.0 mm in diameter after 7 days of incubation on PYG plates at 14°C. Growth occurs at 0–18°C, pH 7.0–9.0, and in the presence of 0–1.0% (w/v) NaCl. The optimum growth temperature is 10–14°C. Positive for catalase, but negative for oxidase. Does not reduce nitrate to nitrite. Does not hydrolyze Tween 80, gelatin, casein, and starch. Hydrolyze esculine. Indole and H_2_S are not formed. Positive for alkaline phosphatase, leucine arylamidase, β-galactosidase, and Voges–Proskauer test. Utilizes the following substances as carbon sources: D-maltose, D-cellobiose, sucrose, D-turanose, D-salicin, α-D-glucose, D-mannose, D-fructose, D-galactose, L-fucose, L-rhamnose, inosine, D-mannitol, D-arabitol, myo-inositol, glycerol, glycyl-L-proline, L-alanine, L-arginine, L-glutamic acid, L-pyroglutamic acid, D-gluconic acid, L-lactic acid, D-malic acid, L-malic acid, bromosuccinic acid, γ-aminobutyric acid, β-hydroxy-D,L-butyric acid, α-keto-butyric acid, acetoacetic acid, propionic acid, acetic acid, and formic acid. Acids are produced from L-arabinose, D-ribose, L-xylose, glucose, mannose, rhamnose, and esculine. The major fatty acids are *anteiso*-C_15:0_, *iso*-C_16:0_, *anteiso*-C_17:0_, *iso*-C_15:0_, and *anteiso*-C_15:1_. The predominant menaquinones include MK-9 and MK-10, with minor amounts of MK-8. The polar lipids are diphosphatidylglycerol, phosphatidylglycerol, one unidentified glycolipid, and one unidentified lipid. The DNA G+C content of the type strain is 64.52%.

The type strain, Hz16^T^ (=CGMCC 1.9272^T^ = NBRC 114048^T^), was isolated from the ice sample obtained from the supraglacial zone of No. 1 glacier in Xinjiang Uygur Autonomous Region, China. The NCBI accession numbers for the 16S rRNA gene and genome sequences are JX949290 and GCA_004403985.1, respectively.

### Description of *Cryobacterium suzukii* sp. nov.

*Cryobacterium suzukii* (su.zu'ki.i, N.L. gen. masc .n. *suzukii* of Suzuki, to honor Professor Ken-ichiro Suzuki who proposed the genus *Cryobacterium*).

Cells are Gram-positive, aerobic, non-motile, rod-shaped, and 1.1–1.6 × 0.5–0.6 μm in size. Cells do not carry any flagella. Colonies are citrine-colored, convex, round, and 1.0 mm in diameter after 7 days of incubation on PYG plates at 14°C. Growth occurs at 0–18°C, pH 7.0–10.0, and in the presence of 0–3.0% (w/v) NaCl. The optimum growth temperature is 10–14°C. Positive for catalase, but negative for oxidase. Reduces nitrate to nitrite. Does not hydrolyze Tween 80, gelatin, casein, and starch. Hydrolyze esculine. Indole and H_2_S are not formed. Positive for alkaline phosphatase, leucine arylamidase, Voges–Proskauer test, and β-galactosidase. Utilizes the following substances as carbon sources: D-cellobiose, sucrose, N-acetyl-D-glucosamine, α-D-glucose, D-fructose, glycerol, glycyl-L-proline, L-alanine, L-arginine, L-glutamic acid, L-pyroglutamic acid, D-gluconic acid, L-lactic acid, D-malic acid, L-malic acid, α-hydroxybutyric acid, β-hydroxy-D,L-butyric acid, α-keto-butyric acid, propionic acid, acetic acid, and formic acid. Acids are produced from L-arabinose, D-ribose, D-xylose, galactose, glucose, fructose, mannose, N-acetylglucosamine, esculine, D-cellobiose, D-maltose, D-saccharose, D-trehalose, and D-turanose. The major fatty acids are *anteiso*-C_15:0_, *anteiso*-C_17:0_, *iso*-C_16:0_, anteiso-C_15:1_, and iso-C_15:0_. The predominant menaquinones include MK-10 and MK-9, with minor amounts of MK-8. The polar lipids are diphosphatidylglycerol, phosphatidylglycerol, one unidentified glycolipid, and one unidentified lipid. The DNA G+C content of the type strain is 64.05%.

The type strain, Sr39^T^ (=CGMCC 1.9276^T^ = NBRC 114032^T^), was isolated from the ice sample obtained from the supraglacial zone of No. 1 glacier in Xinjiang Uygur Autonomous Region, China. The NCBI accession numbers for the 16S rRNA gene and genome sequences are JX949299 and GCA_004403185.1, respectively.

### Description of *Cryobacterium fucosi* sp. nov.

*Cryobacterium fucosi* (fu.co'si. N.L. gen. n. *fucosi*, of fucose).

Cells are Gram-positive, aerobic, motile with a single flagellum, rod-shaped, and 1.0–1.7 × 0.4–0.6 μm in size. Colonies are citrine-colored, convex, round, and 2.0 mm in diameter after 7 days of incubation on PYG plates at 14°C. Growth occurs at 0–24°C, pH 6.0–10.0, and in the presence of 0–2.0% (w/v) NaCl. The optimum growth temperature is 14–20°C. Positive for catalase, but negative for oxidase. Reduces nitrate to nitrite. Does not hydrolyze Tween 80, gelatin, casein, and starch. Hydrolyze esculine. Indole and H_2_S are not formed. Positive for leucine arylamidase, α-glucosidase, arginine dihydrolase, Voges–Proskauer test, and β-galactosidase. Utilizes the following substances as carbon sources: dextrin, D-maltose, D-trehalose, D-cellobiose, gentiobiose, sucrose, D-turanose, α-D-lactose, D-melibiose, D-salicin, N-acetyl-D-glucosamine, α-D-glucose, D-mannose, D-fructose, D-galactose, inosine, D-mannitol, glycerol, D-fructose-6-PO_4_, L-alanine, L-arginine, L-glutamic acid, L-histidine, L-serine, D-gluconic acid, p-hydroxyphenylacetic acid, L-lactic acid, Tween 40, γ-aminobutyric acid, α-hydroxybutyric acid, propionic acid, and acetic acid. Acids are produced from L-arabinose, D-ribose, D-xylose, galactose, glucose, fructose, mannose, mannitol, N-acetylglucosamine, D-cellobiose, D-maltose, D-saccharose, D-trehalose, starch, glycogene, and gentiobiose. The major fatty acids are *anteiso*-C_15:0_, *iso*-C_16:0_, *anteiso*-C_15:1_, and *anteiso*-C_17:0_. The predominant menaquinones include MK-11, MK-12, and MK-10. The polar lipids are diphosphatidylglycerol, phosphatidylglycerol, one unidentified glycolipid, and one unidentified lipid. The DNA G+C content of the type strain is 68.04%.

The type strain, Hh4^T^ (=CGMCC 1.9290^T^ = NBRC 114036^T^), was isolated from the ice sample obtained from the supraglacial zone of No. 1 glacier in Xinjiang Uygur Autonomous Region, China. The NCBI accession numbers for the 16S rRNA gene and genome sequences are JX949272 and GCA_004403515.1, respectively.

### Description of *Cryobacterium frigoriphilum* sp. nov.

*Cryobacterium frigoriphilum* (fri.go.ri'phi.lum. L. neut. n. *frigor*, the cold; N.L. masc. adj. *philus* (from Gr. masc. adj. philos), loving; N.L. neut. adj. *frigoriphilum*, cold-loving).

Cells are Gram-positive, aerobic, non-motile, rod-shaped, and 1.5–1.8 × 0.7–0.9 μm in size. Cells do not carry any flagella. Colonies are naples yellow-colored, convex, round, and 2.0 mm in diameter after 7 days of incubation on PYG plates at 14°C. Growth occurs at 0–18°C, pH 7.0–10.0, and in the presence of 0–3.0% (w/v) NaCl. The optimum growth temperature is 10–14°C. Positive for catalase and oxidase. Reduces nitrate to nitrite. Does not hydrolyze Tween 80, gelatin, casein, and starch. Hydrolyze esculine. Indole and H_2_S are not formed. Positive for alkaline phosphatase, esterase lipase (C8), leucine arylamidase, acid phosphatase, α-glucosidase, β-glucosidase, Voges–Proskauer test, and β-galactosidase. Utilizes the following substances as carbon sources: D-cellobiose, gentiobiose, β-methyl-D-glucoside, D-salicin, α-D-glucose, D-mannose, D-fructose, D-galactose, inosine, D-sorbitol, D-mannitol, D-arabitol, myo-inositol, glycerol, L-alanine, L-pyroglutamic acid, D-galacturonic acid, L-galactonic acid lactone, D-gluconic acid, D-glucuronic acid, L-lactic acid, citric acid, L-malic acid, α-hydroxybutyric acid, β-hydroxy-D,L-butyric acid, propionic acid, and acetic acid. Acids are produced from galactose, mannose, and esculine. The major fatty acids are *anteiso*-C_15:0_, *iso*-C_16:0_, *iso*-C_15:0_, *anteiso*-C_17:0_, and *anteiso*-C_15:1_. The predominant menaquinone includes MK-10, with minor amounts of MK-9 and MK-11. The polar lipids are diphosphatidylglycerol, phosphatidylglycerol, one unidentified glycolipid, and one unidentified lipid. The DNA G+C content of the type strain is 66.74%.

The type strain, Hh14^T^ (=CGMCC 1.9297^T^ = NBRC 114037^T^), was isolated from the ice sample obtained from the supraglacial zone of No. 1 glacier in Xinjiang Uygur Autonomous Region, China. The NCBI accession numbers for the 16S rRNA gene and genome sequences are JX949277 and GCA_004403305.1, respectively.

### Description of *Cryobacterium cryoconiti* sp. nov.

*Cryobacterium cryoconiti* (cry.o.co.ni'ti. N.L. gen. n. *cryoconiti*, isolated from cryoconite).

Cells are Gram-positive, aerobic, motile with a single flagellum, rod-shaped, and 1.5–2.1 × 0.6–0.8 μm in size. Colonies are yellow-colored, convex, round, and 1.0 mm in diameter after 7 days of incubation on PYG plates at 14°C. Growth occurs at 0–24°C, pH 7.0–10.0, and in the presence of 0–4.0% (w/v) NaCl. The optimum growth temperature is 14–20°C. Positive for catalase, but negative for oxidase. Reduces nitrate to nitrite. Does not hydrolyze Tween 80, gelatin, casein, and starch. Hydrolyze esculine. Indole and H_2_S are not formed. Positive for leucine arylamidase, α-glucosidase, Voges–Proskauer test, and β-galactosidase. Utilizes the following substances as carbon sources: dextrin, D-maltose, D-trehalose, D-cellobiose, gentiobiose, sucrose, D-turanose, stachyose, D-raffinose, α-D-lactose, D-melibiose, α-D-glucose, D-mannose, D-fructose, D-galactose, inosine, glycerol, L-alanine, L-arginine, L-glutamic acid, L-histidine, D-gluconic acid, p-hydroxyphenylacetic acid, L-lactic acid, L-malic acid, bromosuccinic acid, Tween 40, γ-aminobutyric acid, α-hydroxybutyric acid, β-hydroxy-D,L-butyric acid, propionic acid, acetic acid, and formic acid. Acids are produced from galactose, glucose, fructose, mannose, D-maltose, D-melibiose, D-saccharose, and D-raffinose. The major fatty acids are *anteiso*-C_15:0_, *iso*-C16:0, C_16:0_, *anteiso*-C_15:1_, *anteiso*-C_17:0_, and *iso*-C_15:1_. The predominant menaquinones include MK-10 and MK-11. The polar lipids are diphosphatidylglycerol, phosphatidylglycerol, one unidentified glycolipid, and one unidentified lipid. The DNA G+C content of the type strain is 67.76%.

The type strain, TMT1-51^T^ (=CGMCC 1.9350^T^ = NBRC 114038^T^), was isolated from the cryoconite sample obtained from the supraglacial zone of Toumingmengke glacier in Gansu province, China. The NCBI accession numbers for the 16S rRNA gene and genome sequences are JX949903 and GCA_004403065.1, respectively.

### Description of *Cryobacterium lyxosi* sp. nov.

*Cryobacterium lyxosi* (ly.xo'si. N.L. gen. n. *lyxosi*, of lyxose).

Cells are Gram-positive, aerobic, motile with a single flagellum, rod-shaped, and 1.2–2.0 × 0.5–0.7 μm in size. Colonies are gold yellow-colored, convex, round, and 1.0 mm in diameter after 7 days of incubation on PYG plates at 14°C. Growth occurs at 0–18°C, pH 7.0–9.0, and in the presence of 0–2.0% (w/v) NaCl. The optimum growth temperature is 10–14°C. Positive for catalase, but negative for oxidase. Does not reduce nitrate to nitrite. Does not hydrolyze Tween 80, gelatin, casein, and starch. Hydrolyze esculine. Indole and H_2_S are not formed. Positive for alkaline phosphatase, leucine arylamidase, α-glucosidase, Voges–Proskauer test, and β-galactosidase. Utilizes the following substances as carbon sources: dextrin, D-maltose, D-trehalose, D-salicin, α-D-glucose, D-mannose, D-fructose, D-galactose, inosine, D-mannitol, D-arabitol, L-pyroglutamic acid, D-gluconic acid, D-saccharic acid, L-lactic acid, D-malic acid, L-malic acid, bromosuccinic acid, acetoacetic acid, propionic acid, acetic acid, and formic acid. Acids are produced from glycerol, glucose, fructose, mannitol, esculine, D-cellobiose, D-saccharose, D-turanose, and D-lyxose. The major fatty acids are *anteiso*-C_15:0_, *iso*-C_16:0_, *anteiso*-C_17:0_, and *anteiso*-C_15:1_. The predominant menaquinone includes MK-10, with minor amounts of MK-11 and MK-9. The polar lipids are diphosphatidylglycerol, phosphatidylglycerol, one unidentified glycolipid, and one unidentified lipid. The DNA G+C content of the type strain is 63.51%.

The type strain, TMT1-1^T^ (=CGMCC 1.9465^T^ = NBRC 113798^T^), was isolated from the cryoconite sample obtained from the supraglacial zone of Toumingmengke glacier in Gansu province, China. The NCBI accession numbers for the 16S rRNA gene and genome sequences are JX949920 and GCA_004403345.1, respectively.

### Description of *Cryobacterium sinapicolor* sp. nov.

*Cryobacterium sinapicolor* (si.na.pi'co.lor. L. fem. n. *sinapis*, mustard; L. masc. n. *color*, color; N.L. neut. adj. *sinapicolor*, mustard-colored).

Cells are Gram-positive, aerobic, non-motile, rod-shaped, and 1.2–1.8 × 0.6–0.8 μm in size. Cells do not carry any flagella. Colonies are mustard-colored, convex, round, and 1.0 mm in diameter after 7 days of incubation on PYG plates at 14°C. Growth occurs at 0–22°C, pH 7.0–10.0, and in the presence of 0–3.0% (w/v) NaCl. The optimum growth temperature is 10–14°C. Positive for catalase, but negative for oxidase. Reduces nitrate to nitrite. Does not hydrolyze Tween 80, gelatin, casein, and starch. Hydrolyze esculine. Indole and H_2_S are not formed. Positive for leucine arylamidase, arginine dihydrolase, Voges–Proskauer test, and β-galactosidase. Utilizes the following substances as carbon sources: dextrin, D-maltose, D-trehalose, D-cellobiose, gentiobiose, sucrose, α-D-lactose, β-methyl-D-glucoside, D-salicin, N-acetyl-D-glucosamine, N-acetyl-β-D-mannosamine, α-D-glucose, D-mannose, D-fructose, D-galactose, L-rhamnose, inosine, glycerol, L-alanine, L-arginine, L-glutamic acid, L-histidine, L-serine, p-hydroxyphenylacetic acid, methyl pyruvate, L-lactic acid, D-malic acid, L-malic acid, bromosuccinic acid, Tween 40, γ-aminobutyric acid, α-hydroxybutyric acid, acetoacetic acid, propionic acid, and acetic acid. Acids are produced from galactose, glucose, fructose, mannose, rhamnose, esculine, D-cellobiose, D-maltose, starch, and glycogene. The major fatty acids are *anteiso*-C_15:0_, *iso*-C_16:0_, *iso*-C_17:1_ ω5*c, anteiso*-C_17:0_, *anteiso*-C_15:1_, and *anteiso*-C_16:0_. The predominant menaquinone includes MK-10, with minor amounts of MK-11 and MK-9. The polar lipids are diphosphatidylglycerol, phosphatidylglycerol, one unidentified glycolipid, and one unidentified lipid. The DNA G+C content of the type strain is 67.20%.

The type strain, TMT1-23-1^T^ (=CGMCC 1.9483^T^ = NBRC 113799^T^), was isolated from the cryoconite sample obtained from the supraglacial zone of Toumingmengke glacier in Gansu province, China. The NCBI accession numbers for the 16S rRNA gene and genome sequences are JX949927 and GCA_004403465.1, respectively.

### Description of *Cryobacterium sandaracinum* sp. nov.

*Cryobacterium sandaracinum* (san.da.ra.ci'num. Gr. masc. adj. *sandarakinos*, orange; N.L. neut. adj. *sandarakinum*, orange).

Cells are Gram-positive, aerobic, motile with a single flagellum, rod-shaped, and 1.3–2.1 × 0.6–0.8 μm in size. Colonies are peach orange-colored, convex, round, and 1.0 mm in diameter after 7 days of incubation on PYG plates at 14°C. Growth occurs at 0–18°C, pH 7.0–10.0, and in the presence of 0–3.0% (w/v) NaCl. The optimum growth temperature is 10–14°C. Positive for catalase, but negative for oxidase. Reduces nitrate to nitrite. Does not hydrolyze Tween 80, gelatin, casein, and starch. Hydrolyze esculine. Indole and H_2_S are not formed. Positive for leucine arylamidase, arginine dihydrolase, citrate utilization, Voges–Proskauer test, and β-galactosidase. Utilizes the following substances as carbon sources: dextrin, D-maltose, D-trehalose, D-cellobiose, sucrose, D-turanose, N-acetyl-D-glucosamine, α-D-glucose, D-mannose, D-fructose, D-galactose, 3-methyl glucose, inosine, D-mannitol, glycerol, L-alanine, L-arginine, L-glutamic acid, D-gluconic acid, p-hydroxyphenylacetic acid, methyl pyruvate, L-lactic acid, L-malic acid, bromosuccinic acid, Tween 40, γ-aminobutyric acid, α-hydroxybutyric acid, acetoacetic acid, and propionic acid. Acids are produced from D-ribose, galactose, glucose, fructose, mannose, methyl-α-D-mannopyranoside, N-acetylglucosamine, and esculine. The major fatty acids are *anteiso*-C_15:0_, *iso*-C_16:0_, *anteiso*-C_17:0_, *anteiso*-C_15:1_, *iso*-C_17:1_ ω5*c, iso*-C_15:1_, and *iso*-C_16:0_. The predominant menaquinone includes MK-10, with minor amounts of MK-11 and MK-9. The polar lipids are diphosphatidylglycerol, phosphatidylglycerol, one unidentified glycolipid, and one unidentified lipid. The DNA G+C content of the type strain is 66.75%.

The type strain, TMT2-16^T^ (=CGMCC 1.9503^T^ = NBRC 114039^T^), was isolated from the cryoconite sample obtained from the supraglacial zone of Toumingmengke glacier in Gansu province, China. The NCBI accession numbers for the 16S rRNA gene and genome sequences are JX949884 and GCA_004403415.1, respectively.

### Description of *Cryobacterium cheniae* sp. nov.

*Cryobacterium cheniae* (chen'i.ae. N.L. gen. fem. n. *cheniae*, of Chen, to honor Professor Wen-Xin Chen, a respected microbiologist, for her great contributions to the investigation and development of Rhizobia resources in China).

Cells are Gram-positive, aerobic, motile with a single flagellum, rod-shaped, and 1.2–2.1 × 0.7–0.8 μm in size. Colonies are yellow-colored, convex, round, and 1.0 mm in diameter after 7 days of incubation on PYG plates at 14°C. Growth occurs at 0–20°C, pH 7.0–9.0, and in the presence of 0–3.0% (w/v) NaCl. The optimum growth temperature is 10–14°C. Positive for catalase, but negative for oxidase. Reduces nitrate to nitrite. Does not hydrolyze Tween 80, gelatin, casein, and starch. Hydrolyze esculine. Indole and H_2_S are not formed. Positive for leucine arylamidase, α-glucosidase, Voges–Proskauer test, and β-galactosidase. Utilizes the following substances as carbon sources: dextrin, D-maltose, D-trehalose, D-cellobiose, sucrose, D-turanose, α-D-lactose, D-salicin, N-acetyl-D-glucosamine, α-D-glucose, D-mannose, D-fructose, D-galactose, inosine, D-sorbitol, D-mannitol, glycerol, L-alanine, L-arginine, L-glutamic acid, L-histidine, L-serine, pectin, D-gluconic acid, p-hydroxyphenylacetic acid, methyl pyruvate, L-lactic acid, L-malic acid, bromosuccinic acid, Tween 40, γ-aminobutyric acid, α-hydroxybutyric acid, α-keto-butyric acid, acetoacetic acid, propionic acid, acetic acid, and formic acid. Acids are produced from galactose, glucose, fructose, mannose, mannitol, sorbitol, N-acetylglucosamine, D-maltose, D-saccharose, and glycogene. The major fatty acids are *anteiso*-C_15:0_, *iso*-C_16:0_, *anteiso*-C_17:0_, *iso*-C_15:1_, *anteiso*-C_15:1_, and *anteiso*-C_16:0_. The predominant menaquinones include MK-10 and MK-11. The polar lipids are diphosphatidylglycerol, phosphatidylglycerol, one unidentified glycolipid, and one unidentified lipid. The DNA G+C content of the type strain is 67.75%.

The type strain, TMT2-48-2^T^ (=CGMCC 1.9517^T^ = NBRC 114040^T^), was isolated from the cryoconite sample obtained from the supraglacial zone of Toumingmengke glacier in Gansu province, China. The NCBI accession numbers for the 16S rRNA gene and genome sequences are JX949892 and GCA_004403395.1, respectively.

### Description of *Cryobacterium shii* sp. nov.

*Cryobacterium shii* (sh.i'i. N.L. gen. n. *shii*, of Shi, to honor Mr. Ya-Feng Shi, a great geographer and glaciologist, the founder of Glaciology in China).

Cells are Gram-positive, aerobic, motile with a single flagellum, rod-shaped, and 1.2–2.1 × 0.8–0.9 μm in size. Colonies are lemon-colored, convex, round, and 2.0 mm in diameter after 7 days of incubation on PYG plates at 14°C. Growth occurs at 0–22°C, pH 6.0–9.0, and in the presence of 0–3.0 % (w/v) NaCl. The optimum growth temperature is 10–14°C. Positive for catalase, but negative for oxidase. Reduces nitrate to nitrite. Does not hydrolyze Tween 80, gelatin, casein, and starch. Hydrolyze esculine. Indole and H_2_S are not formed. Positive for leucine arylamidase, acid phosphatase, naphthol-AS-BI-phosphohydrolase, arginine dihydrolase, Voges–Proskauer test, and β-galactosidase. Utilizes the following substances as carbon sources: dextrin, D-maltose, D-trehalose, D-cellobiose, sucrose, D-turanose, α-D-glucose, D-mannose, D-fructose, D-galactose, inosine, D-glucose-6-PO_4_, D-fructose-6-PO_4_, L-alanine, L-arginine, L-glutamic acid, L-histidine, L-serine, pectin, D-gluconic acid, p-hydroxyphenylacetic acid, methyl pyruvate, D-lactic acid methyl ester, L-lactic acid, D-malic acid, L-malic acid, bromosuccinic acid, Tween 40, γ-aminobutyric acid, α-hydroxybutyric acid, β-hydroxy-D,L-butyric acid, α-keto-butyric acid, acetoacetic acid, propionic acid, acetic acid, and formic acid. Acids are produced from L-arabinose, galactose, glucose, fructose, mannose, esculine, D-maltose, and D-saccharose. The major fatty acids are *anteiso*-C_15:0_, *iso*-C_16:0_, *anteiso*-C_15:1_, *anteiso*-C_17:0_, *iso*-C_15:1_, and *iso*-C_16:0_. The predominant menaquinones include MK-10 and MK-11. The polar lipids are diphosphatidylglycerol, phosphatidylglycerol, one unidentified glycolipid, and one unidentified lipid. The DNA G+C content of the type strain is 68.14%.

The type strain, TMT1-22^T^ (=CGMCC 1.9687^T^ = NBRC 114041^T^), was isolated from the cryoconite sample obtained from the supraglacial zone of Toumingmengke glacier in Gansu province, China. The NCBI accession numbers for the 16S rRNA gene and genome sequences are JX949935 and GCA_004402595.1, respectively.

### Description of *Cryobacterium glucosi* sp. nov.

*Cryobacterium glucosi* (glu.co'si. N.L. gen. n. *glucosi*, of glucose).

Cells are Gram-positive, aerobic, motile with a single flagellum, rod-shaped, and 1.4–2.0 × 0.5–0.6 μm in size. Colonies are cream-colored, convex, round, and 1.0 mm in diameter after 7 days of incubation on PYG plates at 14°C. Growth occurs at 0–26°C, pH 5.0–8.0, and in the presence of 0–3.0% (w/v) NaCl. The optimum growth temperature is 14–20°C. Positive for catalase, but negative for oxidase. Reduces nitrate to nitrite. Does not hydrolyze Tween 80, gelatin, casein, and starch. Hydrolyze esculine. Indole and H_2_S are not formed. Positive for alkaline phosphatase, leucine arylamidase, acid phosphatase, α-galactosidase, β-galactosidase, α*-*glucosidase, β-glucosidase, arginine dihydrolase, Voges–Proskauer test, and fermentation of glucose. Utilizes the following substances as carbon sources: dextrin, D-maltose, D-trehalose, D-cellobiose, sucrose, D-turanose, stachyose, D-raffinose, D-melibiose, β-methyl-D-glucoside, D-salicin, N-acetyl-D-glucosamine, N-acetyl-D-galactosamine, α-D-glucose, D-mannose, D-fructose, D-galactose, L-rhamnose, inosine, D-sorbitol, D-mannitol, glycerol, glycyl-L-proline, L-alanine, L-arginine, L-glutamic acid, L-histidine, pectin, D-galacturonic acid, L-galactonic acid lactone, D-gluconic acid, D-glucuronic acid, p-hydroxyphenylacetic acid, D-lactic acid methyl ester, L-lactic acid, α-ketoglutaric acid, D-malic acid, L-malic acid, Tween 40, γ-aminobutyric acid, α-hydroxybutyric acid, β-hydroxy-D,L-butyric acid, α-keto-butyric acid, acetoacetic acid, propionic acid, and acetic acid. Acids are produced from glycerol, L-arabinose, D-ribose, D-xylose, methyl-β-D-xylopyranoside, galactose, glucose, fructose, mannose, rhamnose, mannitol, sorbitol, N-acetylglucosamine, amygdaline, arbutin, esculine, salicin, D-cellobiose, D-maltose, D-melibiose, D-saccharose, D-trehalose, D-raffinose, and potassium gluconate. The major fatty acids are *anteiso*-C_15:0_, *anteiso*-C_17:0_, and *iso*-C_16:0_. The predominant menaquinones include MK-10 and MK-11, with minor amounts of MK-9. The polar lipids are diphosphatidylglycerol, phosphatidylglycerol, one unidentified glycolipid, and one unidentified lipid. The DNA G+C content of the type strain is 67.68%.

The type strain, MDB1-5^T^ (=CGMCC 1.9741^T^ = NBRC 114042^T^), was isolated from the ice sample obtained from the supraglacial zone of Midui glacier on the Tibetan Plateau, China. The NCBI accession numbers for the 16S rRNA gene and genome sequences are JX949731 and GCA_004402235.1, respectively.

### Description of *Cryobacterium algoritolerans* sp. nov.

*Cryobacterium algoritolerans* (al.go.ri.to'le.rans. L. masc. n. *algor*, the cold; L. pres. part. *tolerans*, tolerating; N.L. part. adj. *algoritolerans*, cold tolerating).

Cells are Gram-positive, aerobic, motile with a single flagellum, rod-shaped, and 1.4–3.0 × 0.5–0.6 μm in size. Colonies are tangerine yellow-colored, convex, round, and 2.0 mm in diameter after 7 days of incubation on PYG plates at 14°C. Growth occurs at 0–24°C, pH 6.0–8.0, and in the presence of 0–3.0% (w/v) NaCl. The optimum growth temperature is 14–20°C. Positive for catalase, but negative for oxidase. Reduces nitrate to nitrite. Does not hydrolyze Tween 80, gelatin, and casein. Hydrolyze starch and esculine. Indole and H_2_S are not formed. Positive for leucine arylamidase, Voges–Proskauer test, and β-galactosidase. Utilizes the following substances as carbon sources: dextrin, D-maltose, D-trehalose, D-cellobiose, gentiobiose, sucrose, D-turanose, D-raffinose, α-D-lactose, D-melibiose, D-salicin, N-acetyl-D-glucosamine, α-D-glucose, D-mannose, D-fructose, D-galactose, inosine, D-mannitol, D-arabitol, glycerol, glycyl-L-proline, L-alanine, L-arginine, L-glutamic acid, L-histidine, L-serine, pectin, D-gluconic acid, p-hydroxyphenylacetic acid, methyl pyruvate, L-lactic acid, L-malic acid, bromosuccinic acid, Tween 40, α-hydroxybutyric acid, β-hydroxy-D,L-butyric acid, α-keto-butyric acid, propionic acid, acetic acid, and formic acid. Acids are produced from glycerol, L-arabinose, D-ribose, galactose, glucose, fructose, mannose, mannitol, N-acetylglucosamine, esculine, D-maltose, D-saccharose, starch, and glycogene. The major fatty acids are *anteiso*-C_15:0_, *iso*-C_16:0_, *anteiso*-C_17:0_, *anteiso*-C_15:1_, *iso*-C_15:1_, and *iso*-C_16:0_. The predominant menaquinones include MK-11 and MK-12, with minor amounts of MK-10. The polar lipids are diphosphatidylglycerol, phosphatidylglycerol, one unidentified glycolipid, and one unidentified lipid. The DNA G+C content of the type strain is 67.01%.

The type strain, MDT1-3^T^ (=CGMCC 1.9782^T^ = NBRC 114043^T^), was isolated from the cryoconite sample obtained from the supraglacial zone of Midui glacier on the Tibetan Plateau, China. The NCBI accession numbers for the 16S rRNA gene and genome sequences are JX949739 and GCA_004402785.1, respectively.

### Description of *Cryobacterium mannosilyticum* sp. nov.

*Cryobacterium mannosilyticum* (man.no.si.ly'ti.cum. N.L. neut. n. *mannosum*, mannose; N.L. masc. adj. *lyticus* (from Gr. masc. adj. lytikos) dissolving; N.L. neut. adj. *mannosilyticum*, mannose dissolving).

Cells are Gram-positive, aerobic, motile with a single flagellum, rod-shaped, and 1.1–3.4 × 0.5–0.7 μm in size. Colonies are yellow-colored, convex, round, and 2.0 mm in diameter after 7 days of incubation on PYG plates at 14°C. Growth occurs at 0–22°C, pH 5.0–9.0, and in the presence of 0–3.0 % (w/v) NaCl. The optimum growth temperature is 10–14°C. Positive for catalase, but negative for oxidase. Reduces nitrate to nitrite. Does not hydrolyze Tween 80, gelatin, casein, and starch. Hydrolyze esculine. Indole and H_2_S are not formed. Positive for alkaline phosphatase, leucine arylamidase, acid phosphatase, α-glucosidase, α-mannosidase, β-galactosidase, and Voges–Proskauer test. Utilizes the following substances as carbon sources: dextrin, D-maltose, D-trehalose, D-cellobiose, gentiobiose, sucrose, D-turanose, N-acetyl-D-glucosamine, α-D-glucose, D-mannose, D-fructose, D-galactose, inosine, D-sorbitol, glycerol, L-arginine, L-glutamic acid, D-gluconic acid, L-lactic acid, L-malic acid, β-hydroxy-D,L-butyric acid, propionic acid, and acetic acid. Acids are produced from glycerol, L-arabinose, D-ribose, D-xylose, galactose, glucose, fructose, mannose, sorbitol, methyl-α-D-mannopyranoside, esculine, D-maltose, D-saccharose, and D-trehalose. The major fatty acids are *anteiso*-C_15:0_, *iso*-C_16:0_, *anteiso*-C_17:0_, *anteiso*-C_16:0_, and *anteiso*-C_15:1_. The predominant menaquinones include MK-11 and MK-12, with minor amounts of MK-10. The polar lipids are diphosphatidylglycerol, phosphatidylglycerol, one unidentified glycolipid, and one unidentified lipid. The DNA G+C content of the type strain is 68.63%.

The type strain, RHLT2-21^T^ (=CGMCC 1.10060^T^ = NBRC 114044^T^), was isolated from the cryoconite sample obtained from the supraglacial zone of Hailuogou glacier in Sichuan province, China. The NCBI accession numbers for the 16S rRNA gene and genome sequences are JX949475 and GCA_004402705.1, respectively.

### Description of *Cryobacterium adonitolivorans* sp. nov.

*Cryobacterium adonitolivorans* (a.do.ni.to.li.vo'rans. N.L. neut. n. *adonitol*, adonitol; L. pres. part. *vorans*, devouring; N.L. part. adj. *adonitolivorans*, adonitol-devouring).

Cells are Gram-positive, aerobic, motile with a single flagellum, rod-shaped, and 0.9–1.7 × 0.7–0.8 μm in size. Colonies are lemon yellow-colored, convex, round, and 2.0 mm in diameter after 7 days of incubation on PYG plates at 14°C. Growth occurs at 0–26°C, pH 6.0–10.0, and in the presence of 0–5.0% (w/v) NaCl. The optimum growth temperature is 14–20°C. Positive for catalase, but negative for oxidase. Reduces nitrate to nitrite. Does not hydrolyze Tween 80, gelatin, casein, and starch. Hydrolyze esculine. Indole and H_2_S are not formed. Positive for alkaline phosphatase, leucine arylamidase, α-glucosidase, β-glucosidase, β-galactosidase, and Voges–Proskauer test. Utilizes the following substances as carbon sources: dextrin, D-maltose, D-trehalose, D-cellobiose, gentiobiose, sucrose, D-turanose, D-salicin, N-acetyl-D-glucosamine, N-acetyl-β-D-mannosamine, α-D-glucose, D-mannose, D-fructose, D-galactose, inosine, D-sorbitol, D-arabitol, glycerol, D-gluconic acid, methyl pyruvate, citric acid, L-malic acid, bromosuccinic acid, propionic acid, and acetic acid. Acids are produced from glycerol, L-arabinose, D-ribose, D-xylose, D-adonitol, galactose, glucose, mannose, mannitol, sorbitol, esculine, D-cellobiose, D-maltose, and D-arabitol. The major fatty acids are *anteiso*-C_15:0_, *iso*-C_16:0_, *anteiso*-C_17:0_, *iso*-C_17:1_ ω5*c, anteiso*-C_15:1_, and *anteiso*-C_16:0_. The predominant menaquinones include MK-10 and MK-9, with minor amounts of MK-11. The polar lipids are diphosphatidylglycerol, phosphatidylglycerol, one unidentified glycolipid, and one unidentified lipid. The DNA G+C content of the type strain is 67.61%.

The type strain, RHLS22-1^T^ (=CGMCC 1.10101^T^ = NBRC 114045^T^), was isolated from the melt water sample obtained from the supraglacial zone of Hailuogou glacier in Sichuan province, China. The NCBI accession numbers for the 16S rRNA gene and genome sequences are JX949476 and GCA_004402695.1, respectively.

### Description of *Cryobacterium algoricola* sp. nov.

*Cryobacterium algoricola* (al.go.ri'co.la. L. masc. n. *algor*, the cold; L. masc./fem. suff. -*cola*, inhabitant, dweller; from L. masc./fem. n. *incola*, dweller; N.L. masc./fem. n. *algoricola*, cold-dweller).

Cells are Gram-positive, aerobic, motile with a single flagellum, rod-shaped, and 0.8–2.3 × 0.5–0.6 μm in size. Colonies are creamy yellow-colored, convex, round, and 1.0 mm in diameter after 7 days of incubation on PYG plates at 14°C. Growth occurs at 0–24°C, pH 6.0–8.0, and in the presence of 0–2.0% (w/v) NaCl. The optimum growth temperature is 14–20°C. Positive for catalase and oxidase. Reduces nitrate to nitrite. Does not hydrolyze Tween 80, gelatin, casein, and starch. Hydrolyze esculine. Indole and H_2_S are not formed. Positive for leucine arylamidase, acid phosphatase, β-galactosidase, α-glucosidase, β-glucosidase, and Voges–Proskauer test. Utilizes the following substances as carbon sources: D-maltose, D-trehalose, D-cellobiose, sucrose, D-turanose, stachyose, D-raffinose, D-melibiose, β-methyl-D-glucoside, D-salicin, N-acetyl-D-glucosamine, α-D-glucose, D-mannose, D-fructose, D-galactose, L-rhamnose, D-mannitol, glycerol, D-gluconic acid, L-lactic acid, L-malic acid, β-hydroxy-D,L-butyric acid, propionic acid, and acetic acid. Acids are produced from L-arabinose, D-xylose, galactose, glucose, fructose, mannose, rhamnose, mannitol, N-acetylglucosamine, arbutin, esculine, salicin, D-cellobiose, D-maltose, D-melibiose, D-saccharose, D-trehalose, and D-raffinose. The major fatty acids are *anteiso*-C_15:0_, *iso*-C_16:0_, *anteiso*-C_15:1_, and anteiso-C_17:0_. The predominant menaquinones include MK-10 and MK-11, with minor amounts of MK-9. The polar lipids are diphosphatidylglycerol, phosphatidylglycerol, one unidentified glycolipid, and one unidentified lipid. The DNA G+C content of the type strain is 67.90%.

The type strain, MDB2-B^T^ (=CGMCC 1.11135^T^ = NBRC 114047^T^), was isolated from the ice sample obtained from the supraglacial zone of Midui glacier on the Tibetan Plateau, China. The NCBI accession numbers for the 16S rRNA gene and genome sequences are JX949747 and GCA_004402485.1, respectively.

### Description of *Cryobacterium tagatosivorans* sp. nov.

*Cryobacterium tagatosivorans* (ta.ga.to.si.vo'rans. N.L. neut. adj. *tagatosum*, tagatose; L. pres. part. *vorans*, eating; N.L. part. adj. *tagatosivorans*, eating tagatose).

Cells are Gram-positive, aerobic, motile with a single flagellum, rod-shaped, and 1.4–3.0 × 0.5–0.6 μm in size. Colonies are creamy yellow-colored, convex, round, and 2.0 mm in diameter after 7 days of incubation on PYG plates at 14°C. Growth occurs at 0–24°C, pH 7.0–10.0, and in the presence of 0–3.0% (w/v) NaCl. The optimum growth temperature is 14–20°C. Positive for catalase and oxidase. Reduces nitrate to nitrite. Does not hydrolyze Tween 80, gelatin, casein, and starch. Hydrolyze esculine. Indole and H_2_S are not formed. Positive for leucine arylamidase, α-glucosidase, Voges–Proskauer test, and β-galactosidase. Utilizes the following substances as carbon sources: D-maltose, D-cellobiose, stachyose, D-raffinose, D-melibiose, N-acetyl-D-glucosamine, N-acetyl-D-galactosamine, α-D-glucose, D-mannose, D-fructose, D-galactose, L-fucose, L-rhamnose, D-sorbitol, D-mannitol, D-arabitol, glycerol, L-alanine, L-arginine, L-glutamic acid, L-histidine, L-pyroglutamic acid, L-serine, D-gluconic acid, L-lactic acid, D-malic acid, L-malic acid, γ-aminobutyric acid, β-hydroxy-D,L-butyric acid, propionic acid, and acetic acid. Acids are produced from glycerol, glucose, fructose, rhamnose, mannitol, sorbitol, esculine, D-cellobiose, D-maltose, D-lyxose, D-tagatose, L-fucose, and D-arabitol. The major fatty acids are *anteiso*-C_15:0_, *anteiso*-C_15:1_, *iso*-C_15:0_, *iso*-C_16:0_, and *anteiso*-C_17:0_. The predominant menaquinones include MK-11 and MK-12, with minor amounts of MK-10. The polar lipids are diphosphatidylglycerol, phosphatidylglycerol, one unidentified glycolipid, and one unidentified lipid. The DNA G+C content of the type strain is 68.28%.

The type strain, Sr47^T^ (=CGMCC 1.11221^T^ = NBRC 114033^T^), was isolated from the ice sample obtained from the supraglacial zone of No. 1 glacier in Xinjiang Uygur Autonomous Region, China. The NCBI accession numbers for the 16S rRNA gene and genome sequences are JX949300 and GCA_004402215.1, respectively.

### Description of *Cryobacterium glaciale* sp. nov.

*Cryobacterium glaciale* (gla.cia'le. L. neut. adj. *glaciale*, icy).

Cells are Gram-positive, aerobic, motile with a single flagellum, rod-shaped, and 1.4–3.3 × 0.6–0.7 μm in size. Colonies are creamy yellow-colored, convex, round, and 2.0 mm in diameter after 7 days of incubation on PYG plates at 14°C. Growth occurs at 0–20°C, pH 6.0–10.0, and in the presence of 0–4.0% (w/v) NaCl. The optimum growth temperature is 10–14°C. Positive for catalase, but negative for oxidase. Reduces nitrate to nitrite. Does not hydrolyze Tween 80, gelatin, casein, and starch. Hydrolyze esculine. Indole and H_2_S are not formed. Positive for alkaline phosphatase, leucine arylamidase, acid phosphatase, α-glucosidase, β-galactosidase, arginine dihydrolase, citrate utilization, and Voges–Proskauer test. Utilizes the following substances as carbon sources: D-maltose, D-trehalose, D-cellobiose, gentiobiose, sucrose, D-turanose, stachyose, β-methyl-D-glucoside, D-salicin, α-D-glucose, D-mannose, D-fructose, L-rhamnose, inosine, D-sorbitol, D-mannitol, D-arabitol, myo-inositol, glycerol, L-alanine, L-arginine, pectin, D-galacturonic acid, L-galactonic acid lactone, D-gluconic acid, D-glucuronic acid, L-lactic acid, L-malic acid, Tween 40, γ-aminobutyric acid, α-hydroxybutyric acid, acetoacetic acid, propionic acid, and acetic acid. Acids are produced from glycerol, glucose, fructose, mannose, rhamnose, esculine, and D-cellobiose. The major fatty acids are *anteiso*-C_15:0_, *iso*-C_16:0_, *anteiso*-C_17:0_, *iso*-C_15:0_, and *anteiso*-C_15:1_. The predominant menaquinones include MK-10 and MK-9, with minor amounts of MK-11. The polar lipids are diphosphatidylglycerol, phosphatidylglycerol, one unidentified glycolipid, and one unidentified lipid. The DNA G+C content of the type strain is 65.39%.

The type strain, HLT2-23^T^ (=CGMCC 1.11085^T^ = NBRC 114046^T^), was isolated from the ice sample obtained from the supraglacial zone of Hailuogou glacier in Sichuan province, China. The NCBI accession numbers for the 16S rRNA gene and genome sequences are JX949477 and GCA_004402535.1, respectively.

## Data availability statement

The datasets presented in this study can be found in online repositories. The names of the repository/repositories and accession number(s) can be found in the article/Supplementary material.

## Author contributions

Y-HX and QL designed the project, analyzed the data, collected and purified the strains, and wrote the manuscript. QL and L-LY performed bioinformatic analysis and experiments on phenotypic and chemotaxonomic characteristics. QL performed fatty acids analysis. All authors contributed to the article and approved the submitted version.
