# Supplementary material for: Diversity of the genus Cryobacterium and proposal of 19 novel species isolated from glaciers
Source: Front Microbiol. 2023 Mar 20;14:1115168. doi: 10.3389/fmicb.2023.1115168 (PMC10067761; doi:10.3389/fmicb.2023.1115168)
Supplement: Supplementary file 2 [file Data_Sheet_2.DOCX]

Supplementary Material

# Supplementary Figures and Tables

## Supplementary Tables

**Supplementary Table 2.** Closest BLASTn matches for the almost complete 16S rRNA gene sequences of the 19 *Cryobacterium* strains.

| **Strain** | **Nearest phylogenetic neighbor** | **Sequence similarity(%)** |
| --- | --- | --- |
| Sr54^T^ | *Cryobacterium* *ruanii* Sr36^T^ | 99.65 |
| Sr59^T^ | *Cryobacterium* *soli* GCJ02^T^ | 99.93 |
| Hz16^T^ | *Cryobacterium* *ruanii* Sr36^T^ | 99.86 |
| Sr39^T^ | *Cryobacterium* *ruanii* Sr36^T^ | 99.93 |
| Hh4^T^ | *Cryobacterium* *breve* TMT4-23^T^ | 99.36 |
| Hh14^T^ | *Cryobacterium* *roopkundense* RuGl7^T^ | 98.51 |
| TMT1-51^T^ | *Cryobacterium* *breve* TMT4-23^T^ | 99.79 |
| TMT1-1^T^ | *Cryobacterium* *luteum* Hh15^T^ | 99.86 |
| TMT1-23-1^T^ | *Cryobacterium* *breve* TMT4-23^T^ | 99.28 |
| TMT2-16^T^ | *Cryobacterium* *breve* TMT4-23^T^ | 99.42 |
| TMT2-48-2^T^ | *Cryobacterium* *breve* TMT4-23^T^ | 99.36 |
| TMT1-22^T^ | *Cryobacterium* *breve* TMT4-23^T^ | 99.50 |
| MDB1-5^T^ | *Cryobacterium* *breve* TMT4-23^T^ | 98.85 |
| MDT1-3^T^ | *Cryobacterium* *breve* TMT4-23^T^ | 99.57 |
| RHLT2-21^T^ | *Cryobacterium* *breve* TMT4-23^T^ | 99.86 |
| RHLS22-1^T^ | *Cryobacterium* *soli* GCJ02^T^ | 99.57 |
| MDB2-B^T^ | *Cryobacterium* *breve* TMT4-23^T^ | 98.76 |
| Sr47^T^ | *Cryobacterium* *psychrotolerans* 0549^T^ | 100.00 |
| HLT2-23^T^ | *Cryobacterium* *flavum* Hh8^T^ | 99.79 |

**Supplementary Table 3**. Phenotypic characteristics of the type strains of 19 novel *Cryobacterium* species.

| **Strain** | **Tmax (°C)** | **pH for growth** | **NaCl Tolerance （%, w/v）** | **Colony color** | **Cell size （μm）** | **Flagellum** |
| --- | --- | --- | --- | --- | --- | --- |
| Sr54^T^ | 18 | 6-10 | 0-3 | gold | 1.5-1.7×0.7-0.8 | - |
| Sr59^T^ | 26 | 6-10 | 0-5 | citrine | 1.3-2.4×0.7-0.8 | + |
| Hz16^T^ | 18 | 7-9 | 0-1 | golden poppy | 1.1-1.7×0.7-0.9 | + |
| Sr39^T^ | 18 | 7-10 | 0-3 | citrine | 1.1-1.6×0.5-0.6 | - |
| Hh4^T^ | 24 | 6-10 | 0-2 | citrine | 1.0-1.7×0.4-0.6 | + |
| Hh14^T^ | 18 | 7-10 | 0-3 | naples yellow | 1.5-1.8×0.7-0.9 | - |
| TMT1-51^T^ | 24 | 7-10 | 0-4 | yellow | 1.5-2.1×0.6-0.8 | + |
| TMT1-1^T^ | 18 | 7-9 | 0-2 | golden yellow | 1.2-2.0×0.5-0.7 | + |
| TMT1-23-1^T^ | 22 | 7-10 | 0-3 | mustard | 1.2-1.8×0.6-0.8 | - |
| TMT2-16^T^ | 18 | 7-10 | 0-3 | orange | 1.3-2.1×0.6-0.8 | + |
| TMT2-48-2^T^ | 20 | 7-9 | 0-3 | yellow | 1.2-2.1×0.7-0.8 | + |
| TMT1-22^T^ | 22 | 6-9 | 0-3 | lemon | 1.2-2.1×0.8-0.9 | + |
| MDB1-5^T^ | 26 | 5-8 | 0-3 | cream | 1.4-2.0×0.5-0.6 | + |
| MDT1-3^T^ | 24 | 6-8 | 0-3 | tangerine yellow | 1.4-3.0×0.5-0.6 | + |
| RHLT2-21^T^ | 22 | 5-9 | 0-3 | yellow | 1.1-3.4×0.5-0.7 | + |
| RHLS22-1^T^ | 26 | 6-10 | 0-5 | lemon yellow | 0.9-1.7×0.7-0.8 | + |
| MDB2-B^T^ | 24 | 6-8 | 0-2 | creamy yellow | 0.8-2.3×0.5-0.6 | + |
| Sr47^T^ | 24 | 7-10 | 0-4 | creamy yellow | 1.4-3.0×0.5-0.6 | + |
| HLT2-23^T^ | 20 | 6-10 | 0-4 | creamy yellow | 1.4-3.3×0.6-0.7 | + |

## Supplementary Figures


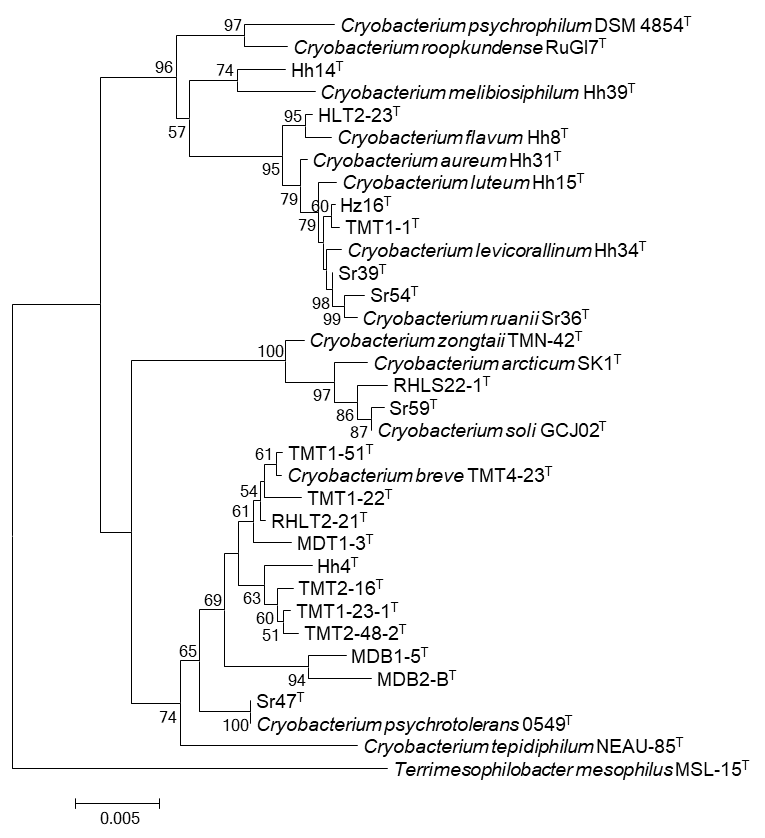


**Supplementary Figure 1.** Phylogenetic tree of genus Cryobacterium based on the comparison of 16S rRNA gene sequences using the neighbor-joining method. Bootstrap values (> 50 %) based on 1,000 replicates are shown at the branch nodes. Bar, 0.005 substitutions per nucleotide positions.


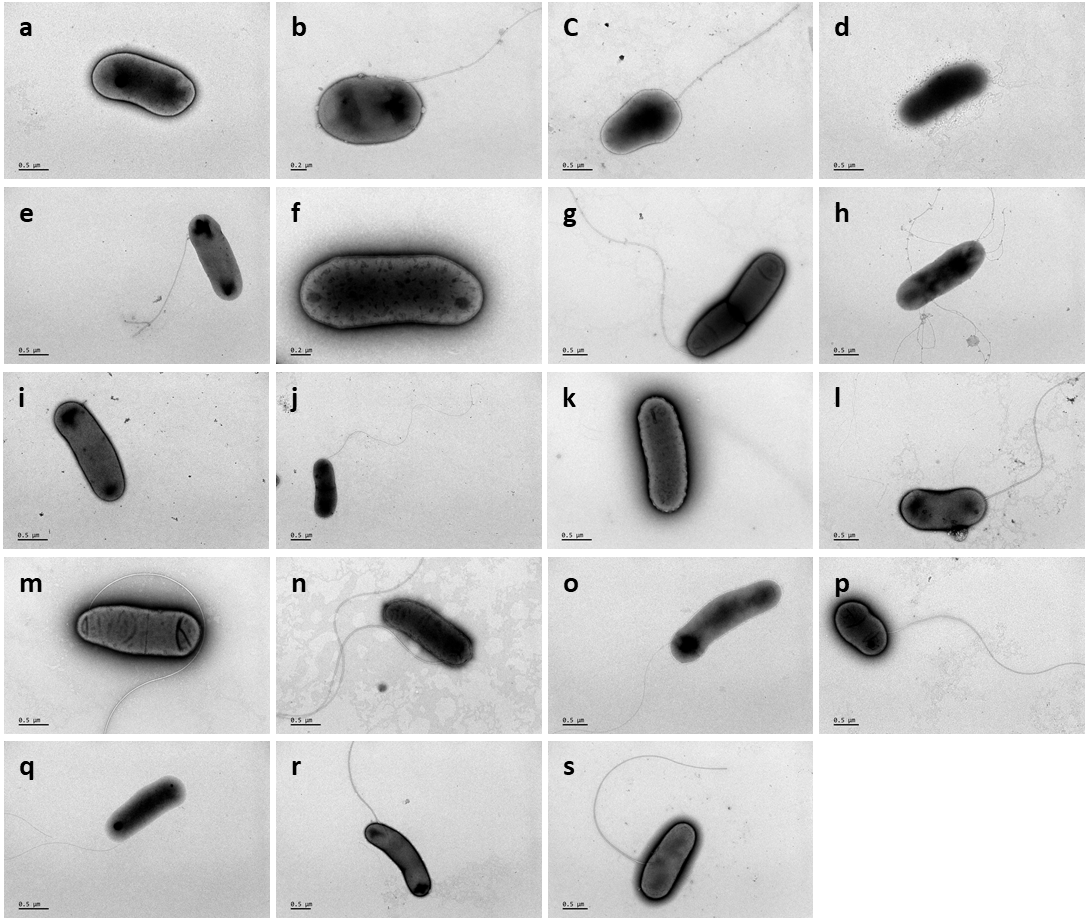


**Supplementary Figure 2**. Transmission electron micrograph of negatively-stained cells of the type strains of 19 novel *Cryobacterium* species. a, *Cryobacterium* *serini* Sr54^T^; b, *Cryobacterium* *lactosi* Sr59^T^; c, *Cryobacterium* *gelidum* Hz16^T^; d, *Cryobacterium* *suzukii* Sr39^T^; e, *Cryobacterium* *fucosi* Hh4^T^; f, *Cryobacterium* *frigoriphilum* Hh14^T^; g, *Cryobacterium* *cryoconiti* TMT1-51^T^; h, *Cryobacterium* *lyxosi* TMT1-1^T^; i, *Cryobacterium* *sinapicolor* TMT1-23-1^T^; j, *Cryobacterium* *sandaracinum* TMT2-16^T^; k, *Cryobacterium* *cheniae* TMT2-48-2^T^; l, *Cryobacterium* *shii* TMT1-22^T^; m, *Cryobacterium* *glucosi* MDB1-5^T^; n, *Cryobacterium* *algoritolerans* MDT1-3^T^; o, *Cryobacterium* *mannosilyticum* RHLT2-21^T^; p, *Cryobacterium* *adonitolivorans* RHLS22-1^T^; q, *Cryobacterium* *algoricola* MDB2-B^T^; r, *Cryobacterium* *tagatosivorans* Sr47^T^; s, *Cryobacterium* *glaciale* HLT2-23^T^.
